# Sequential Nucleophilic Aromatic Substitution Reactions of Activated Halogens

**DOI:** 10.3390/ijms25158162

**Published:** 2024-07-26

**Authors:** M. John Plater, William T. A. Harrison

**Affiliations:** Department of Chemistry, University of Aberdeen, Meston Walk, Aberdeen AB24 3UE, UK

**Keywords:** nucleophilic substitution, sulphur–nitrogen chemistry, activated halogen, fluorine, nitro group

## Abstract

Building blocks have been identified that can be functionalised by sequential nucleophilic aromatic substitution. Some examples are reported that involve the formation of cyclic benzodioxin and phenoxathiine derivatives from 4,5-difluoro-1,2-dinitrobenzene, racemic quinoxaline thioethers, and sulfones from 2,3-dichloroquinoxaline and (2-aminophenylethane)-2,5-dithiophenyl-4-nitrobenzene from 1-(2-aminophenylethane)-2-fluoro-4,5-dinitrobenzene. Four X-ray single-crystal structure determinations are reported, two of which show short intermolecular N–O^…^N “π hole” contacts.

## 1. Introduction

**Figure 1:**
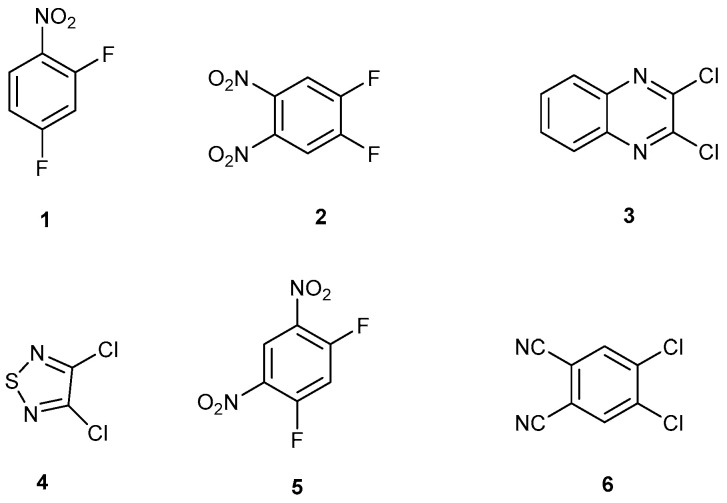
Dihalogenated molecules that can undergo two sequential nucleophilic substitution reactions.

Halogens activated to nucleophilic aromatic substitution are scaffold building blocks for functional materials and heterocycles with interesting properties (Figure 1). Some examples are listed here: 2,4-difluoronitrobenzene, **1**, reacts with one or two different amines which can give porous organic materials with a flexible framework; [1,2,3] and references cited herein. 4,5-Difluoro-1,2-dinitrobenzene, **2**, reacts with 1,2-disubstituted amines, alcohols, and thiols, forming N-heteroacenes, phenoxazines, and phenothiazines; [4] and references cited herein. 2,3-Dichloroquinoxaline reacts with different nucleophiles to give novel pharmaceutical building blocks [5,6]. 2,3-Dichlorothiadiazole, **4**, is representative of a general field of research known as sulphur–nitrogen (S–N) chemistry pioneered by Charles W Rees [7,8,9,10]. 1,3-Difluoro-4,6-dinitrobenzene, **5**, a building block for Marfey’s reagent, is useful in peptide chemistry [11,12]. 1,2-Dichloro-4,5-dicyanobenzene, **6**, is a building block for substituted phthalocyanines [13]. 

**Figure 2:**
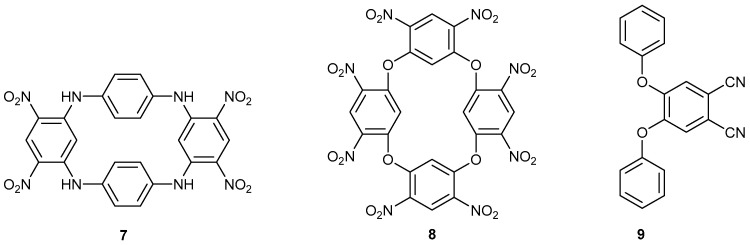
Products **7** and **8**, or product **9**, that can be made from compounds **5** or **6**, respectively.

Figure 2 shows some products that can be made from compounds **5** or **6,** respectively. Compound **7** is an easily made cyclophane [14], and macrocycle **8** is a precursor to an energetic substance [15]. Phthalonitrile **9** is a phthalocyanine precursor for optical limiting in polished polycarbonate discs [16].

## 2. Results and Discussion

**Figure 3:**
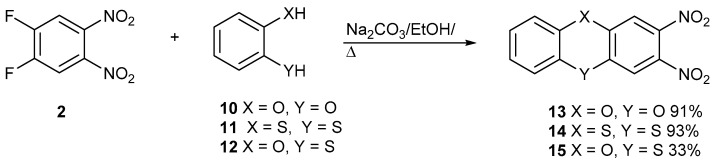
The synthesis of dioxin **13**, dithiin **14**, and phenoxathiine **15**.

New studies are being reported with building block **2** and catechol, **10**, dithiocatechol, **11**, and 2-hydroxythiophenol, **12**, to make dioxin **13**, dithiin **14**, and phenoxathiin **15**, respectively (Figure 3). In the supplementary section charts for proton and carbon NMR data for all new compounds are reported. 

**Figure 4:**
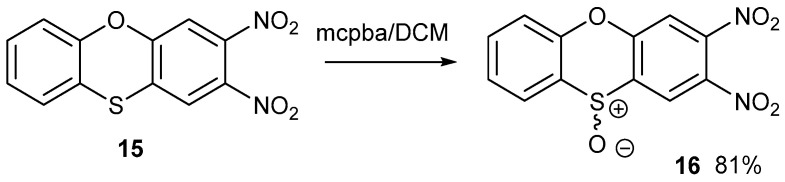
The oxidation of phenoxathiin **15** to form a racemic S-oxide **16**.

The two nitro groups activate both halogens to sequential nucleophilic displacement by phenoxide and thiolate anions. The yields are good for these syntheses. The synthesis of compound **14** was reported by us previously, but it is included here for comparison with other data. Figure 4 shows the synthesis of racemic S-oxide, **16**. Although the yield was good, the yield for the synthesis of the precursor heterocycle **15** was poor, which restricted the amount of material made. Further detailed studies were only carried out on compound **14**. 

Compound **15** was oxidised with *meta*-chloroperbenzoic (mcpba) acid in DCM at room temperature to S-oxide **16** on a small scale. Owing to the racemic nature of this S-oxide, it was difficult to obtain good crystals, possibly due to the disorder of the S-oxide grouping. The oxidation of cyclic sulphide **15** to sulfoxide **16** stopped at the sulfoxide without over-oxidation to a sulfone.

X-ray single-crystal structure determinations were carried out on compounds **13**–**15**.

**Figure 5:**
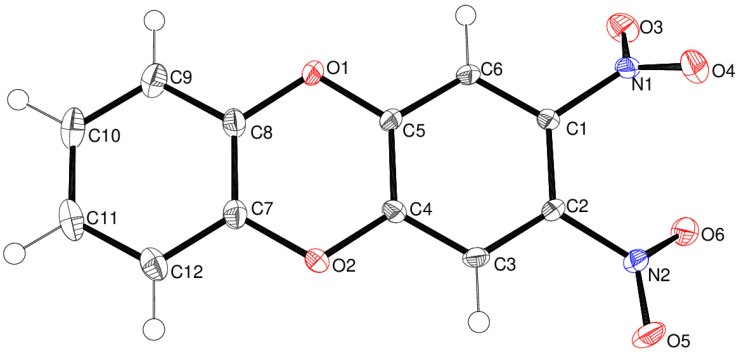
The molecular structure of compound **13** showing 50% displacement ellipsoids.

Compound **13** crystallises in the triclinic space group *P*$\bar{1}$ with one molecule in the asymmetric unit (Figure 5). The C1–C6 and C7–C12 rings are slightly puckered by 5.54 (6)°, which correlates with the fact that the dioxin ring is a very shallow boat with atoms CO1 and O2 displaced from the best plane of C4/C5/C7/C8 by –0.0722 (15) and –0.0917 (14) Å, respectively. The C5–O1–C8 and C4–O2–C7 bond angles are 115.48 (8) and 115.55 (8)°, respectively. Both nitro groups are twisted from their attached C1–C6 ring, by 54.43 (9)° for N1/O3/O4 and 27.21 (7)° for N2/O5/O6. In the extended structure of compound **13,** the molecules are linked by weak C–H^…^O interactions, but there are no identified short contacts involving the nitro groups. In unsubstituted dibenzo-*p*-dioxin [17] C_12_H_8_O_2_, all the atoms lie on a crystallographic mirror plane, and the C–O–C bond angle is 116.4°. 

**Figure 6:**
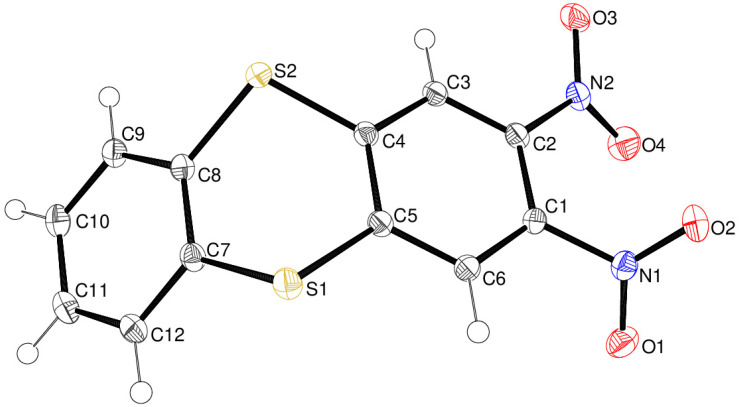
The molecular structure of compound **14** showing 50% displacement ellipsoids.

Compound **14** displays monoclinic crystal symmetry (space group *P*2_1_/*n*) with one molecule in the asymmetric unit (Figure 6). The outer C1–C6 and C7–C12 rings are substantially puckered by 50.89 (3)°, and the dithiin ring is well described as a boat with atoms S1 and S2 displaced from the best plane of C4/C5/C7/C8 (r.m.s. deviation = 0.001 Å) by 0.6979 (13) and 0.6723 (12) Å, respectively. The C5–S1–C7 and C4–S2–C8 bond angles are 99.84 (4) and 100.31 (5)°, respectively (mean = 100.1°); these angles are much smaller than the corresponding bond angles (via O atoms) in compound **13**, which is consistent with the trend that bond angles decrease for more polarisable atoms in the same group of the periodic table [18]. In the structure [19] of unsubstituted thianthrene, C_12_H_8_S_2_, the dihedral angle between the aromatic rings is 50.5°, and the mean C–S–C bond angle is 100.2°. Both nitro groups in compound **14** are twisted from their attached C1–C6 ring, by 53.72 (8)° for N1/O1/O2 and 27.41 (8)° for N2/O3/O4; these dihedral angles are notably similar to the corresponding values for **13**. 

**Figure 7:**
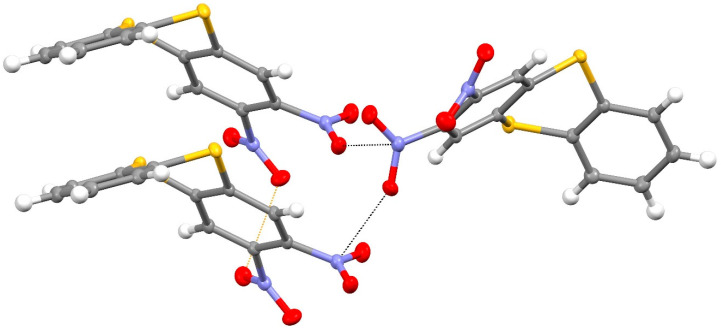
Short N–O^…^N contacts (black dashed lines) in the extended structure of compound **14**, which generate [010] chains. Yellow is sulfur, red is oxygen and blue is nitrogen.

In the extended structure of compound **14**, the molecules are linked by weak C–H^…^O and C–H^…^S interactions as well as unusual short N–O^…^N “π hole” contacts [20] between symmetry-related N1-nitro groups (Figure 7) with O1^…^N1^i^ = 2.8969 (13) Å and N1–O1^…^N1^i^ = 145.90 (7)° (i = ½–*x*, *y*–½, ½–*z*), which generate [010] chains with adjacent molecules related by the 2_1_ screw axis (the van der Waals separation of O and N is about 3.07 Å). The dihedral angle between the N1 and N1^i^ nitro groups is 69.70 (7)°. 

**Figure 8:**
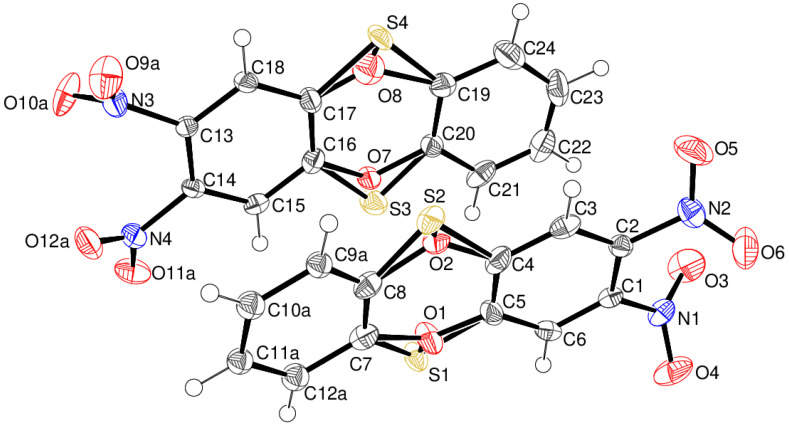
The molecular structure of compound **15** showing 50% displacement ellipsoids. Both disorder components of the oxathiine rings are shown, but for clarity, only the major disorder components of the C7–C12 ring and N3 and N4 nitro groups are drawn.

Compound **15** crystallises with two molecules (A containing C1 and B containing C13) in the asymmetric unit (Figure 8) of space group *P*$\bar{1}$, both of which display disorder: in molecule A, ‘flip’ (~180° rotational) disorder about the long axis of the molecule of the O and S atoms of the oxathiine ring [occupancy of S1/O2 = 0.493 (5); occupancy of S2/O1 = 0.507 (5)] occurs as well as positional disorder of the C7–C12 ring. The B molecule also shows flip disorder [occupancy of S3/O8 = 0.180 (4); occupancy of S4/O7 = 0.820 (4)] as well as disorder of the oxygen atoms of the N3 and N4 nitro groups in 0.715 (4):0.285 (4) and 0.720 (6): 0.280 (6) ratios, respectively. The extensive disorder complicates the detailed interpretation of the ring conformations, but it may be stated that the molecules are close to planar, with dihedral angles between the C1–C6 and C7–C12 (major component) rings of 12.29 (12)° and C13–C18 and C19–C24 of 4.20 (8)°. In the extended structure of compound **15**, the molecules are linked by weak C–H^…^O bonds, but there are no short contacts involving the nitro groups. 

**Figure 9:**
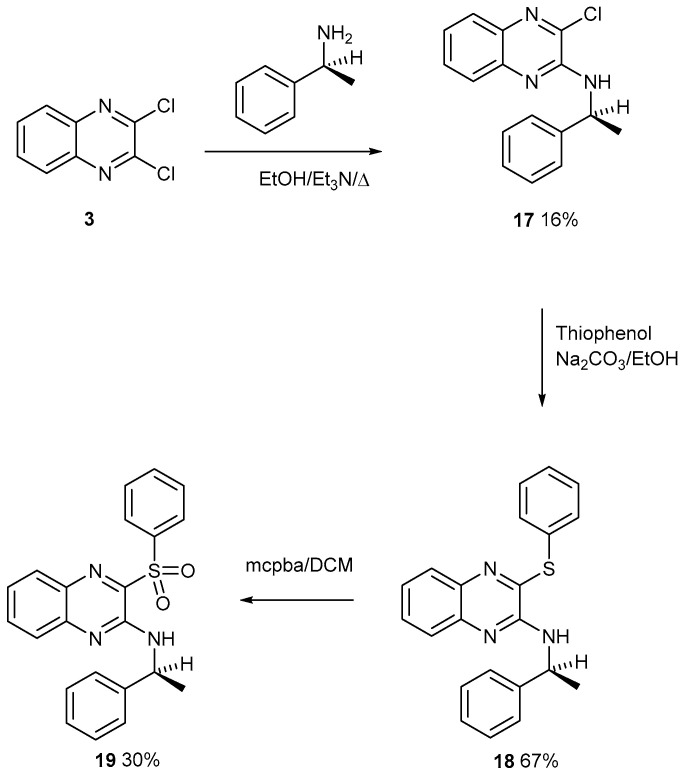
The formation of sulfone **19** from acrylic sulphide **18** with mcpba.

The building blocks in Figure 1 are all commercially available, so more were investigated. Each chlorine atom of compound **3** is activated by a pyridine-type nitrogen atom. Compound **3** was reacted with a cheap optically pure (*S*) amine by a nucleophilic aromatic substitution reaction to give product **17** (Figure 9). This was reacted with a thiolate anion which is a strong nucleophile owing to its size and polarisability. Product **18** was treated with mcpba with a view to make the mono S-oxide (not drawn). Instead, the product was identified, with a clean mass spectrum, as sulfone **19** shown in Figure 9. Here, and in our previous studies, the oxidation of a cyclic sulphide stopped at the mono S-oxide, but here the acyclic sulphide smoothly converted to the sulfone. The oxidation of a cyclic sulfoxide is presumed to have a higher energy barrier. Previously, we discussed the enantiomeric fractionation of chiral sulfoxides from a silica column, which has been reported, but not for single enantiomers. The sulfoxide and sulfone have similar *R*_f_ values, which might complicate the fractionation. No X-ray single-crystal structures were obtained on compounds **17**–**19**. The asymmetry possibly inhibits the growth of good crystals. 

**Figure 10:**
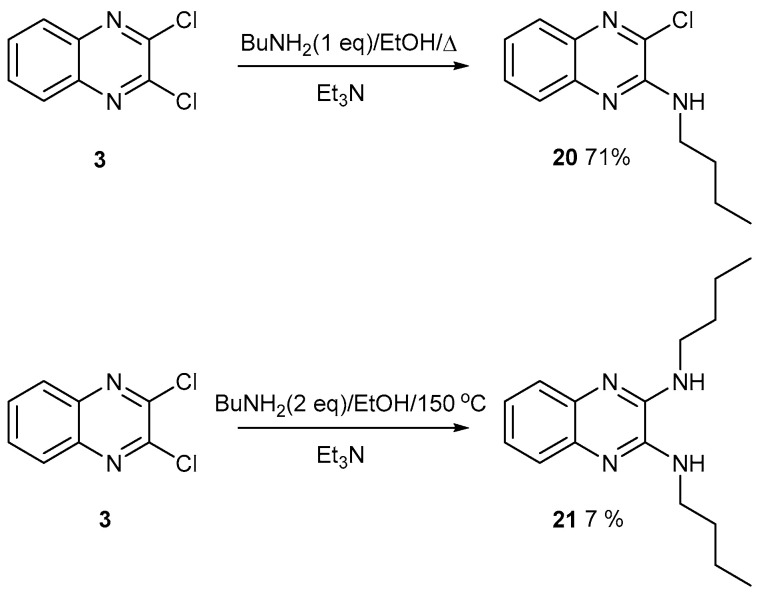
Displacement of one or two chlorine atoms from 2,3-dichloroquinoxaline **3** with butylamine.

Either one [10] or two chlorine atoms can be displaced from 2,3-dichloroquinoxaline, **3**, with butylamine (Figure 10). Since the second Cl atom is harder to displace, and ideally requires a thiolate anion as a nucleophile, the reaction was carried out in a Parr PTFE-lined pressure vessel at a higher temperature of 150 °C. The product forms in low yield but was more polar and harder to purify. In our studies on potential porous organic materials, we found that polar compounds were harder to purify, so the polarity was reduced with butylamine substituents [1,2]. For example, if butylamine was replaced with methyl, ethyl, or propylamine, no products were isolated with smaller compounds.

All four substituents on compound **2** are activated because each nitro group activates the other one as well as a *para*-fluorine atom (Figure 3) [21,22]. An X-ray single-crystal structure determination was carried out on compound **24**.

**Figure 11:**
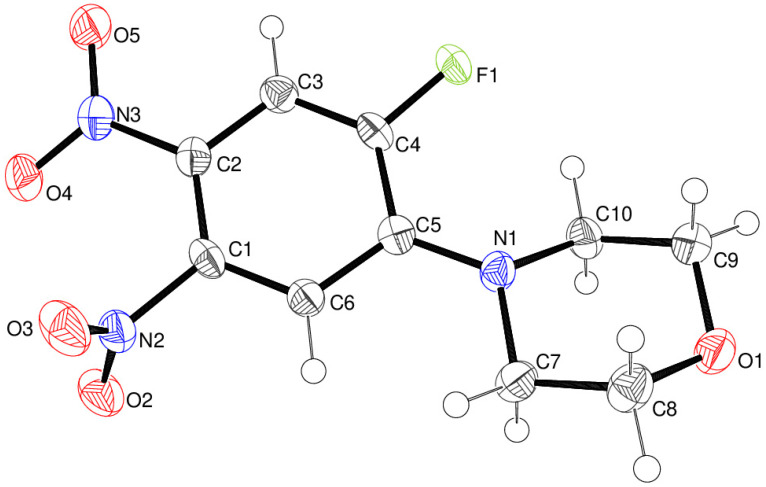
The molecular structure of compound **24** showing 50% displacement ellipsoids.

Compound **24** crystallises in space group *Pca*2_1_ with one molecule in the asymmetric unit (Figure 11). The morpholine ring adopts a normal chair conformation [displacements of N1 and O1 from C7 to C10 = –0.652 (4) and 0.653 (4) Å, respectively] with the exocyclic N1–C5 bond in an equatorial conformation, although the bond angle sum at N1 of 354° is suggestive of a tendency towards *sp*^2^ hybridisation. The dihedral angle between the rings (all atoms) is 24.33 (11)°. The N3/O4/O5 nitro group lies close to the plane of the C1–C6 ring [dihedral angle = 9.2 (4)°], whereas N2/O2/O3 is substantially twisted [82.57 (14)°]. This can be explained by conjugation of the N3 nitro group with atom N1 via the aromatic ring (i.e., a quinoid C=N^+^ resonance form); the C2–N3 bond length [1.441 (4) Å] is clearly shorter than C1–N2 [1.474 (4) Å], and the mean of C1–C6 and C3–C4 [1.368 Å] is shorter than the mean of C1–C2, C2–C3, C4–C5, and C5–C6 [1.401 Å].

**Figure 12:**
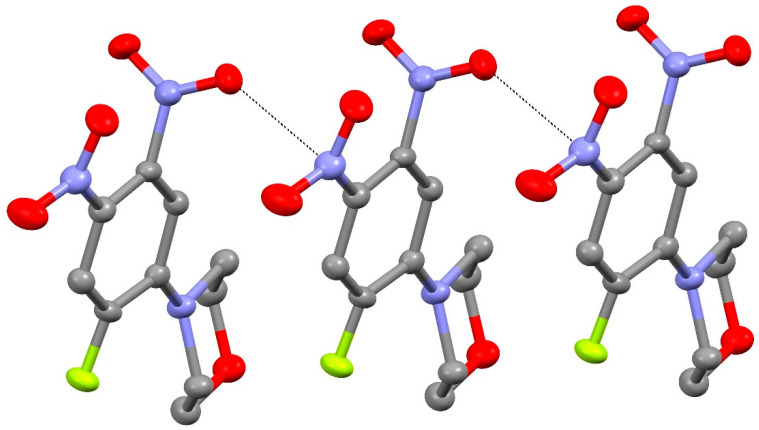
Short O^…^N contacts (black dashed lines) in the extended structure of compound **24**, which generate [010] chains.

The extended structure of **24** features weak C–H^…^O and C–H^…^F interactions as well as notably short N2–O2^…^N3^ii^ (ii = *x*, 1+*y*, *z*) contacts (Figure 12), which lead to [010] chains with adjacent molecules related by translation: the O^…^N separation is 2.839 (3) Å, the N–O^…^N angle is 132.20 (19)°, and the dihedral angle between the N2 and N3^ii^ nitro groups is 81.5 (3)°.

**Figure 13:**
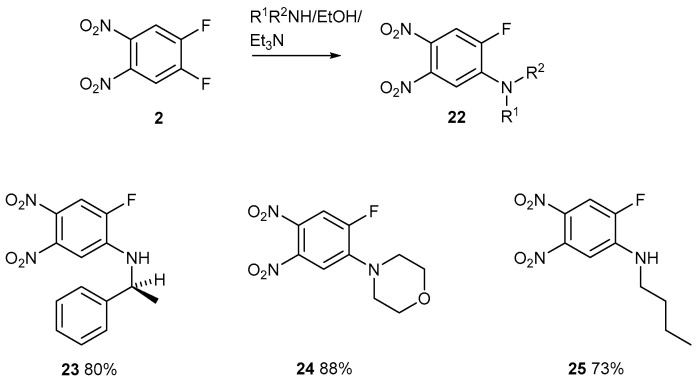
The displacement of one activated fluorine atom from compound **2** with different amines.

**Figure 14:**
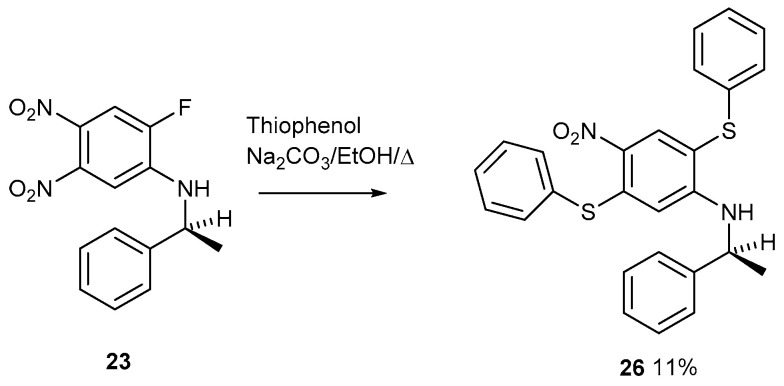
The sequential displacement of an activated fluorine atom followed by an activated nitro group on compound **23**.

Although these reactions are feasible because one fluorine atom is easily displaced (Figure 13), the next reaction posed considerable difficulty (Figure 14). 

A TLC plate run after the reaction work-up showed three spots running close together. The middle spot was eventually isolated as the pure product after running a multiple number of long (12″ × 1″) columns. The data fits for structure **26** are shown. The fluorine atom must be the first group to be displaced; otherwise, two thioethers could not be formed. The nitro group conjugated to the amine is deactivated by this conjugation. After the fluorine atom, the more reactive nitro group is displaced next. This reaction is interesting and represents a formal addition–substitution of diphenyldisulfide across the 1 and 4 positions of a benzene ring, which we believe to be a new reaction. The yield is only about 10%, but the product is pure with good data. 

## 3. Material and Methods

IR spectra were recorded on a diamond-attenuated total reflection (ATR) Fourier transform infrared (FTIR) spectrometer, Nicolet Summit Everest, (Thermo Fischer Scientific, 1 Ashley Road, Altrincham, Cheshire, England); Ultraviolet (UV) spectra were recorded using a Perkin Elmer Lambda 25 UV–Vis spectrometer with EtOH as the solvent (LAS Chalfont, Seer Green, Beaconsfield, England); The term sh means shoulder. ^1^H and ^13^C nuclear magnetic resonance (NMR) spectra were recorded at 400 and 100.5 MHz, respectively, using a Bruker 400 spectrometer (Bruker UK Ltd., Welland House, Westwood Business Park, Coventry, England); Chemical shifts, δ, are given in ppm and measured by comparison with the residual solvent. Coupling constants, *J*, are given in Hz. High-resolution mass spectra were obtained at the University of Wales, Swansea, using an Atmospheric Solids Analysis Probe (ASAP) (positive mode) instrument: Xevo G2-S ASAP (Waters Ltd., Stamford Avenue, Altrincham Road, Wilmslow, England); Melting points were determined on a Cole-Palmer MP-200D Stuart digital melting point microscope (CamLab, Norman Way Ind. Estate, Over, Cambridge, England).

2,3-Dinitrodibenzo-[1,4]-dioxin **13**

4,5-Difluoro-1,2-dinitrobenzene **2** (300 mg, 1.47 mmol) in EtOH (30 mL) was mixed with catechol (162 mg, 1.47 mmol) and Na_2_CO_3_ (2.0 g) and then stirred at 75 °C for 20 h. The mixture was added to water (200 mL) and allowed to stand for 1 h. This was filtered and air-dried for 2 days to give a bright yellow precipitate of the *title compound* (365 mg, 91%) as yellow crystals, 205–206 mp °C (from dichloromethane/light petroleum ether). λ_max_ (EtOH)/nm 279 (log ε 3.6) and 369 sh (3.0); ν_max_ (diamond) (cm^–1^) 3062 w, 1626 w, 1594 w, 1537 s, 1505 s, 1486 s, 1340 s, 1305 s, 1290 s, 1199 s, 890 s, 873 s, 821 s, 765 s, 750 s, and 450 w; δ_H_ (400 MHz; CDCl_3_), 7.05–7.10 (4H, m) and 7.89 (2H, s); δ_C_ (100.1 MHz; CDCl_3_) 114.0, 117, 126.3, 138.5, 140.2, and 145.4; *m/z* (Orbitrap ASAP) 275.0300 (M^+^ + H, 100%) C_12_H_6_N_2_O_6_H requires 275.0304.

2,3-Dinitrophenoxathiine **15**

4,5-Difluoro-1,2-dinitrobenzene **2** (300 mg, 1.47 mmol) in EtOH (30 mL) was mixed with 2-hydroxythiophenol (185 mg, 1.47 mmol) and Na_2_CO_3_ (2.0 g) and then stirred at 75 °C for 20 h. After cooling, the mixture was added to water (200 mL), extracted with DCM (100 mL) twice, followed by drying of the combined extracts over MgSO_4_. The product was purified by chromatography on silica (twice). Elution with dichloromethane/light petroleum ether (40:60) gave the *title compound* (145 mg, 34%) as pure red crystals, 144–145 mp °C (from dichloromethane/light petroleum ether). Alternatively, after dilution of the reaction with water (200 mL) and treatment with 5 M HCl (20 mL), the product was filtered to give the *title compound* (140 mg, 33%). λ_max_ (EtOH)/nm 233 (log ε 3.6), 284 (3.6) and 400 sh (3.0); ν_max_ (diamond) (cm^–1^) 3100 w, 1528 s, 1468 s, 1443 s, 1383 w, 1337 s, 1291 s, 1273 s, 1237 s, 893 s, 841 s, 749 s, 718 s, 682 w, and 447 w; δ_H_ (400 MHz; CDCl_3_) 7.10 (1H, d, *J* = 8.0), 7.18 (1H, t, *J* = 8.0), 7.27–7.33 (2H, m), 7.87 (1H, s) and 8.27 (1H, s); δ_C_ (100.1 MHz; CDCl_3_) 114.8, 116.1, 118.4, 124.6, 127.0, 127.5, 127.6, 129.9, 138.5, 142.0, 149.4, and 154.2; *m/z* (Orbitrap ASAP) 291.0077 (M^+^ + H, 100%) C_12_H_6_N_2_O_5_SH requires 291.0076.

2,3-Dinitrophenoxathiin-S-oxide 16

2,3-Dinitrophenoxathiin **15** (47 mg, 0.16 mmol) in DCM (30 mL) was treated with *meta*-chloroperbenzoic acid (mcpba) (47 mg, 0.32 mmol) for 24 h at rt. The DCM layer was diluted with more DCM (70 mL), extracted with dilute KOH) (4 pellets of KOH dissolved in 200 mL of H_2_O), dried over MgSO_4_, and then concentrated and purified by chromatography on silica. DCM eluted the *title compound* (40 mg, 81%) as a white powder, mp 191–192 °C (from dichloromethane/light petroleum ether). λ_max_ (EtOH)/nm 221 (log ε 4.2) and 299 (3.8); ν_max_ (diamond) (cm^–1^) 1013 w, 1591 w, 1533 s, 1463 s, 1443 s, 1395 s, 1275 s, 1232 s, 1051 s, 840 s, 762 s, and 540 s; δ_H_ (400 MHz; CDCl_3_) 7.59 (1H, t, *J* = 8.0), 7.65 (1H, *J* = 8.0), 7.84 (1H, *J* = 8.0) and 8.14 (1H, *J* = 8.0), 8.50 (1H, s), and 9.18 (1H, s); δ_C_ (100.1 MHz; CDCl_3_) 117.1, 119.2, 123.6, 127.2, 128.0, 130.4, 131.6, 135.5, 136.9, 145.8, 147.9, and 152.5; *m/z* (Orbitrap ASAP) 307.0021 (M^+^ + H, 100%) C_12_H_6_N_2_O_6_S_2_H requires 307.0025.

2-Aminophenylethane-3-chloroquinoxaline **17**

2,3-Dichloroquinoxaline **3** (300 mg, 1.51 mmol), (S)-phenylethylamine (364 mg, 3.0 mmol), and Et_3_N (152 mg, 1.5 mmol) in EtOH (30 mL) were heated under reflux for 48 h. After cooling, the mixture was diluted with water (200 mL) and left to stand for 1 h. The white precipitate was filtered off through a sinter and the product was collected as one fibrous mat. This was purified by chromatography on flash silica. DCM eluted the *title compound* (67 mg, 16%) as a clear oil (from dichloromethane/light petroleum ether). λ_max_ (EtOH)/nm 237 (log ε 4.0) and 358 (3.8); ν_max_ (diamond) (cm^–1^) 3428 w, 1571 s, 1555 s, 1508 s, 1490 s, 1451 s, 1405 s, 1268 s, 1242 s, 1215 s, 1125 w, 1054 vs, 1015 w, 937 w, 829 w, 754 vs, 695 vs and 595 s; δ_H_ (400 MHz; CDCl_3_) 1.70 (3H, d, *J* = 14.0), 5.48 (1H, q, *J* = 8.0), 5.83 (1H, d, *J* = 8.0), 7.30 (1H, dd, *J* = 8.0 and 8.0), 7.37–7.42 (3H, m), 7.49 (2H, d, *J* = 8.0), 7.59 (1H, t, *J* = 8.0), 7.72 (1H, d, *J* = 8.0) and 7.80 (1H, d, J = 8.0); δ_C_ (100.1 MHz; CDCl_3_) 22.1, 50.5, 125.1, 126.2, 126.4, 127.4, 127.9, 128.7, 130.0, 136.5, 137.7, 141.3, 143.5 and 147.1; *m/z* (Orbitrap ASAP) 284.0955 (M^+^ + H, 100%) C_16_H_14_N_3_ClH requires 284.0955.

2-Aminophenylethane-3-thiophenylquinoxaline **18**

2-Aminophenylethane-3-chloroquinoxaline **17** (40 mg, 0.14 mmol) and thiophenol (64 mg, 0.58 mmol) in EtOH (30 mL) with Na_2_CO_3_ (0.5 g) was heated under reflux with stirring for 18 h. Upon cooling, it was diluted in water (200 mL), extracted with DCM (100 mL), dried over MgSO_4_, and filtered. The product was purified by chromatography on flash silica. The column was made up with light petroleum ether 40–60. DCM eluted front by-products and then the *title compound* (34 mg, 67%) as a colourless oil (from dichloromethane/light petroleum ether). λ_max_ (EtOH)/nm 226 (log ε 3.9) and 362 (3.7); ν_max_ (diamond) (cm^–1^) 3407 w, 1542 s, 1505 s, 1439 s, 1405 w, 1266 w, 1240 w, 1214 w, 1124 w, 1054 s, 1022 w, 756 s, 742 s, 696 s, 686 s, 608 w, 596 s and 537 w; δ_H_ (400 MHz; CDCl_3_) 1.60 (3H, d, *J* = 8.0), 5.47 (1H, q, *J* = 8.0), 5.68 (1H, d, *J* = 8.0), 7.25–7.29 (1H, m), 7.31–7.36 (5H, m), 7.40–7.41(3H, m), 7.50–7.54 (3H, m), 7.69 (1H, d, *J* = 8.0) and 7.75 (1H, d, *J* = 8.0); δ_C_ (100.1 MHz; CDCl_3_) 22.3, 50.4, 124.5, 126.1, 126.3, 127.2, 128.3, 128.6, 129.5, 130.1, 132.7, 137.4, 141.1, 143.9, 144.2 and 148.8 (two peaks are overlapping; peak at 129.5 is asymmetric); *m/z* (Orbitrap ASAP) 358.1374 (M^+^ + H, 100%) C_22_H_19_N_3_SH requires 358.1378.

2-Aminophenylethane-3-thiophenylquinoxaline sulfone **19**

2-Aminophenylethane-3-thiophenylquinoxaline **18** (35 mg, 0.098 mmol) in DCM (30 mL) was treated with mcpba (34 mg, 0.197 mmol) and stirred for 18 h at rt. The DCM layer was extracted with water (100 mL) and made basic with 5 pellets of KOH. It was then extracted with water (100 mL), dried with MgSO_4_, and filtered. The product was purified by chromatography on a silica column 12 inches in length and a 1-inch-wide outer diameter. DCM eluted the *title compound* (11 mg, 30%) as a yellow solid, mp 111–113 °C (from dichloromethane/light petroleum ether). λ_max_ (EtOH)/nm 254 (log ε 4.0), 305 (3.1) and 400 (3.1); ν_max_ (diamond) (cm^–1^) 3394 w, 3378 w, 1542 s, 1523 s, 1444 s, 1360 s, 1315 s, 1301 s, 11,250 s, 1137 s, 1087 s, 1056 s, 752 s, 732 s, 714 s, 700 s, 684 s, 631 s, 620 s, 565 s, 549 s, 475 s and 429 s; δ_H_ (400 MHz; CDCl_3_) 1.71 (3H, d, *J* = 8.0), 5.49 (1H, q, *J* = 8.0), 7.28 (1H, t, *J* = 8.0 and 8.0), 7.37 (3H, t, *J* = 8.0 and 8.0), 7.46 (2H, d, *J* = 8.0), 7.57 (2H, t, *J* = 8.0 and 8.0), 7.61–7.63 (2H, m), 7.67 (2H, t, *J* = 8.0 and 8.0), 7.82 (1H, d, *J* = 8.0), 8.10 (2H, d, J = 8.0); δ_C_ (100.1 MHz; CDCl_3_) 22.5, 50.2, 125.4, 126.2, 126.3, 127.2, 128.7, 128.8, 129.2, 129.8, 132.9, 134.3, 135.5, 138.6, 141.5, 143.6, 143.8 and 147.5; *m*/*z* (Orbitrap ASAP) 390.1279 (M^+^ + H, 100%) C_22_H_19_N_3_SO_2_H requires 390.1276.

2-Butylamino-3-chloroquinoxaline **20**

2,3-Dichloroquinoxaline **3** (200 mg, 1.0 mmol) in EtOH (30 mL) was treated with butylamine (147 mg, 2.0 mmol) and triethylamine (203 mg, 2.0 mmol). The mixture was heated at 75 °C for 18 h. After cooling, the mixture was diluted with water (200 mL), extracted with DCM (100 mL), back-extracted with water (100 mL), dried with MgSO_4_, and evaporated to dryness. The product was purified by flash chromatography on silica. Elution with DCM gave the *title compound* (167 mg, 71%) as colourless crystals, mp 44–45 °C (from dichloromethane/light petroleum ether). λ_max_ (EtOH)/nm 347 (log ε 3.5); ν_max_ (diamond) (cm^–1^) 3433 s, 2962 s, 2926 s, 2858 s, 1575 s, 1555 s, 1510 s, 1460 s, 1405 s, 1291 s, 1085 s, 1048 s, 829 s, 765 s, 756 s and 587 s; δ_H_ (400 MHz; CDCl_3_) 0.90 (3H, t, *J* = 8.0), 1.38 (2H, s, *J* = 8.0), 1.61 (2H, q, *J* = 8.0), 3.50 (2H, q, *J* = 8.0), 5.44 (1H, s, br), 7.28 (1H, t, *J* = 8.0 and 8.0), 7.47(1H, t, *J* = 8.0 and 8.0), 7.62 (1H, d, *J* = 8.0) and 7.70 (1H, d, *J* = 8.0); δ_C_ (100.1 MHz; CDCl_3_) 13.7, 20.1, 31.0, 41.1, 124.7, 125.8, 127.9, 129.9, 136.3, 138.0, 141.2 and 148.3; *m/z* (Orbitrap ASAP) 236.0956 (M^+^ + H, 100%) C_12_H_14_N_3_ClH requires 236.0954.

2,3-bis(Butylamino)quinoxaline **21**

2,3-Dichloroquinoxaline **3** (200 mg, 1.0 mmol) in EtOH (10 mL) was treated with butylamine (147 mg, 2.0 mmol) and triethylamine (203 mg, 2.0 mmol) in a 23 mL PTFE-lined Parr Pressure Vessel. The vessel was heated at 150 °C for 18 h and then cooled. The mixture was diluted with water (200 mL), extracted with DCM (100 mL), back-extracted with water (100 mL), dried with MgSO_4_, and evaporated to dryness. The product was purified by flash chromatography on silica. Elution with Et_2_O/DCM (10:90) gave the *title compound* (18 mg, 7%) as colourless crystals, mp 111–112 °C (from dichloromethane/light petroleum ether). λ_max_ (EtOH)/nm 350 (log ε 3.8); ν_max_ (diamond) (cm^–1^) 3337 s, 2957 s, 2928 s, 2860 s, 1595 s, 1555 s, 1502 s, 1455 s, 1322 s, 1203 s, 1143 s, 747 vs, 604 s, 462 s; δ_H_ (400 MHz; CDCl_3_) 0.97 (6H, t, *J* = 8.0), 1.47 (4H, s, *J* = 8.0), 1.69 (4H, q, *J* = 8.0), 3.58 (4H, t, *J* = 8.0), 4.62 (2H, s br), 7.32 (2H, dd, *J* = 4.0 and 4.0) and 7.67 (2H, dd, *J* = 4.0); δ_C_ (100.1 MHz; CDCl_3_) 13.6, 20.3, 31.3, 41.6, 124.5, 125.5, 136.7 and 144.3; *m/z* (Orbitrap ASAP) 273.2078 (M^+^ + H, 100%) C_16_H_24_N_4_H requires 273.2079.

1-(2-Aminophenylethane)-2-fluoro-4,5-dinitrobenzene **23**

4,5-Difluoro-1,2-dinitrobenzene **2** (300 mg, 1.47 mmol) and 2-aminophenylethane (178 mg, 1.47 mmol) in EtOH (30 mL) were treated with Et_3_N (149 mg, 1.47 mmol) and refluxed for 24 h. Upon cooling, the mixture was diluted with water (200 mL), extracted with DCM (100 mL), separated, and dried over MgSO_4_. After filtration and evaporation, the mixture was purified by chromatography on silica. DCM eluted the *title compound* (360 mg, 80%) as a yellow solid, mp 143–144 °C (from dichloromethane/light petroleum ether). λ_max_ (EtOH)/nm 228 (log ε 3.5) and 378 (3.4); ν_max_ (diamond) (cm^–1^) 3373 w, 1617 s, 1541 s, 1508 s, 1475 s, 1450 s, 1386 s, 1314 s, 1292 s, 1247 s, 1209 s, 1184 s, 1119 s, 1040 s, 882 s, 845 s, 820 s, 801 s, 746 s, 697 s, 657 s, 615 s, 592 s, 533 s and 452 s; δ_H_ (400 MHz; CDCl_3_) 1.67 (3H, d, *J* = 8.0), 4.63 (1H, q, *J* = 8.0), 5.26 (1H, s), 6.63 (1H, d, *J* = 4.0), 7.30–7.43 (5H, m) and 7.79 (1H, d, *J* = 8.0); δ_C_ (100.1 MHz; CDCl_3_) 24.3, 53.4, 106.5, 112.2 (1C, d, *J* = 20), 125.5, 128.2, 129.4, 141.0 (1C, d, *J* = 5), 141.4, 142.9 and 148.8 (1CF, d, *J* = 220); *m/z* (Orbitrap ASAP) 306.0890 (M^+^ + H, 100%) C_14_H_12_N_3_O_4_FH requires 306.0890.

1-Fluoro-2-morpholino-4,5-dinitrobenzene **24**

4,5-Difluoro-1,2-dinitrobenzene **2** (500 mg, 2.45 mmol), morpholine (213 mg, 2.45 mmol), and Et_3_N (248 mg, 2.45 mmol) in EtOH (30 mL) were refluxed for 18 h. After cooling, the reaction was diluted with water (200 mL), left standing for 5 min, and filtered, which worked well. The filtrate was yellow. The product was a single pure spot by TLC. It was air-dried to give the *title compound* (587 mg, 88%) as a yellow solid, mp 167–168 °C (from dichloromethane/light petroleum ether). λ_max_ (EtOH)/nm 380 (log ε 3.2); ν_max_ (diamond) (cm^–1^) 1609 s, 1526 s, 1504 s, 1368 s, 1320 s, 1304 s, 1250 s, 1206 s, 1111 s, 1066 w, 1011 s, 922 s, 870 s, 841 s, 804 s, 750 s, 719 s, 658 s, 631 s, 596 s, 559 s and 424 w; δ_H_ (400 MHz; CDCl_3_) 3.38 (4H, t, *J* = 5.0), 3.90 (4H, t, *J* = 5.0), 7.16 (1H, d, *J* = 4.0) and 7.77 (1H, d, *J* = 8.0); δ_C_ (151.0 MHz; CDCl_3_) 49.7, 66.3, 112.9, 114.5 (1C, d, *J* _C-F_ = 24.0), 132.9, 141.6, 144.2 (1C, d, *J-C* = 24.0) and 153.0 (1C, d, *J* _C-F_ = 172.0) ; *m/z* (Orbitrap ASAP) 272.0679 (M^+^ + H, 100%) C_10_H_10_N_3_O_5_FH requires 272.0683.

1-Fluoro-2-butylamino-4,5-dinitrobenzene **25**

4,5-Difluoro-1,2-dinitrobenzene **2** (500 mg, 2.45 mmol), butylamine (179 mg, 2.45 mmol), and Et_3_N (248 mg, 2.45 mmol) in EtOH (30 mL) were refluxed for 18 h. After cooling, the reaction was diluted with water (200 mL), treated with cHCl (2 mL), left standing for 2 h, and filtered, which worked well until the end. It was air-dried to give the *title compound* (458 mg, 73%) as a yellow solid, mp 95–96 °C (from dichloromethane/light petroleum ether). λ_max_ (EtOH)/nm 382 (log ε 3.4); ν_max_ (diamond) (cm^–1^) 3341 s, 1617 s, 1542 s, 1504 s, 1435 w, 1392 w, 1369 w, 1297 s, 1190 s, 1077 s, 882 s, 806 s, 7703 s, 655 s and 587 s; δ_H_ (400 MHz; CDCl_3_) 1.01 (3H, d, *J* = 7.0), 1.47 (2H, sextet, *J* = 7.0), 1.69 (2H, quintet, *J* = 7.0), 3.3 (2H, m), 4.9 (1H, s, br NH), 6.81 (1H, d, *J-C* = 12.0) and 7.76 (1H, d, *J-C* = 12.0); δ_C_ (100.1 MHz; CDCl_3_) 13.6, 20.1, 30.7, 42.8, 105.1 (1C, d_J-C_ = 4.0), 112.2 (1C, d_J-C_ = 23), 128.0 (1C, d_J-C_ = 10.0), 142.4 (1C, d_J-C_ = 10.0), 143.5, 148.7 (1C, d_J-C_ = 240.0); *m/z* 258.0889 (Orbitrap ASAP) (M^+^ + H, 100%) C_10_H_12_N_3_O_4_FH requires 258.0890.

1-(2-Aminophenylethane)-2,5-dithiophenyl-4-nitrobenzene **26**

1-(2-Aminophenylethane)-2-fluoro-4,5-dinitrobenzene **23** (100 mg, 0.327 mmol), thiophenol (36 mg, 0.327 mmol), and Na_2_CO_3_ (500 mg) in EtOH (30 mL) were heated under reflux with stirring for 24 h. After cooling, the reaction was diluted with water (200 mL), extracted with DCM (100 mL), dried over MgSO_4_, and filtered. After evaporation, the mixture was purified by chromatography on silica. DCM eluted the *title compound* (16 mg, 11%) as a yellow solid, mp 59–60 °C (from dichloromethane/light petroleum ether). Multiple numbers of columns were required to obtain pure product owing to close running spots thought to be diphenyldisulfide and starting material. λ_max_ (EtOH)/nm 240 (log ε 4.0), 297 (3.95) and 373 (3.85); ν_max_ (diamond) (cm^–1^) 1575 s, 1544 s, 1509 s, 1473 s, 1438 s, 1294 vs, 1247 vs, 1118 s, 1073 s, 1021 s, 999 s, 967 s, 907 s, 831 s, 737 vs, 687 vs, 551 s and 429 s; δ_H_ (400 MHz; CDCl_3_) 1.27 (3H, d, *J* = 8.0), 3.92 (1H, q, *J* = 8.0), 6.51 (2H, d, *J* = 8.0), 7.12 (2H, t, *J* = 8.0 and 8.0), 7.17–7.21 (3H, m), 7.26 (1H, d, *J* = 8.0), 7.29–7.30 (4H, m), 7.42–7.43 (3H, m), 7.52–7.55 (1H, m) and 8.57 (1H, s); δ_C_ (100.1 MHz; CDCl_3_) 24.3, 52.8, 107.9, 112.0, 125.3, 126.7, 127.2, 127.6, 128.7, 129.3, 129.8, 130.1, 130.6, 134.1, 134.7, 135.6, 136.2, 142.3, 145.2 and 150.6; *m/z* (Orbitrap ASAP) 459.1199 (M^+^ + H, 100%) C_26_H_22_N_2_O_2_S_2_H requires 459.1201; 369.1071 (M^+^ + H, 100%) C_20_H_17_N_2_O_2_SF requires 369.1062 

### Crystal Structure Determinations

The crystal structures of **13**, **14**, **15**, and **24** (all recrystallised from the mixed solvents of dichloromethane/light petroleum ether) were established using intensity data collected at 100 K on a Rigaku CCD diffractometer using either Mo Kα radiation (**13**, **14,** and **15**) or Cu Kα radiation (**24**). The structures were routinely solved by dual-space methods using SHELXT [23], and the structural models were completed and optimised by refinement against |*F*|^2^ with SHELXL-2019 [24]. Extensive disorder was found for **15**. The H atoms were placed in idealised locations (C–H = 0.95–0.98 Å) and refined as riding atoms with *U*_iso_(H) = 1.2*U*_eq_(carrier). Full details of the structures and refinements are available in the deposited cifs.

- Crystal data for compound **13** C_12_H_6_N_2_O_6_, yellow plate, 0.19 × 0.09 × 0.03 mm, *M*_r_ = 274.19, triclinic, space group *P*$\bar{1}$ (No. 2), *a* = 7.8050 (2) Å, *b* = 8.1532 (3) Å, *c* = 8.9461 (2) Å, α = 97.688 (2)°, β = 99.428 (2)°, γ = 94.297 (2)°, *V* = 553.82 (3) Å^3^, *Z* = 2, Mo Kα radiation (λ = 0.71073 Å),*T* = 100 K, μ = 0.136 mm^–1^, ρ_calc_ = 1.644 g cm^–3^, 26,948 reflections measured (4.7 ≤ 2θ ≤ 61.0°), 2919 unique (*R*_Int_ = 0.040), *R*(*F*) = 0.039 [2919 reflections with *I* > 2σ(*I*)], *wR*(*F*^2^) = 0.113 (all data), Δρ_min,max_ (*e* Å^–3^) = –0.29, +0.47, CCDC deposition number 2362025.
- Crystal data for compound **14** C_12_H_6_N_2_O_4_S_2_, yellow plate, 0.12 × 0.07 ×0.01 mm, *M*_r_ = 306.31, monoclinic, space group *P*2_1_/*n* (No. 14), *a* = 16.4666 (3) Å, *b* = 4.17460 (10) Å, *c* = 17.5585 (4) Å, β = 93.833 (2)°, *V* = 1204.30 (5) Å^3^, *Z* = 4, Mo Kα radiation (λ = 0.71073 Å), *T* = 100 K, μ = 0.457 mm^–1^, ρ_calc_ = 1.689 g cm^–3^, 52,290 reflections measured (4.7 ≤ 2θ ≤ 76.2°), 6238 unique (*R*_Int_ = 0.035), *R*(*F*) = 0.037 [4630 reflections with *I* > 2σ(*I*)], *wR*(*F*^2^) = 0.098 (all data), Δρ_min,max_ (*e* Å^–3^) = –0.24, +0.61, CCDC deposition number 2362026.
- Crystal data for compound **15** C_12_H_6_N_2_O_5_S, orange rod, 0.44 × 0.15 × 0.07 mm, *M*_r_ = 290.25, triclinic, space group *P*$\bar{1}$ (No. 2), *a* = 7.89759 (13) Å, *b* = 11.40431 (17) Å, *c* = 14.0329 (2) Å, α = 70.1769 (13)°, β = 87.4953 (12)°, γ = 79.3130 (13)°, *V* = 1168.13 (3) Å^3^, *Z* = 4, Mo Kα radiation (λ = 0.71073 Å), *T* = 100 K, μ = 0.300 mm^–1^, ρ_calc_ = 1.650 g cm^–3^, 113,652 reflections measured (3.1 ≤ 2θ ≤ 61.0°), 7132 unique (*R*_Int_ = 0.056), *R*(*F*) = 0.078 [6713 reflections with *I* > 2σ(*I*)], *wR*(*F*^2^) = 0.166 (all data), Δρ_min,max_ (*e* Å^–3^) = –0.74, +0.89, CCDC deposition number 2362027.
- Crystal data for compound **24** C_10_H_10_FN_3_O_5_, yellow slab, 0.05 × 0.04 × 0.02 mm, *M*_r_ = 271.21, orthorhombic, space group *Pca*2_1_ (No. 29), *a* = 18.1738 (6) Å, *b* = 4.60825 (14) Å, *c* = 13.3823 (5) Å, *V* = 1120.76 (7) Å^3^, *Z* = 4, Cu Kα radiation (λ = 1.54184 Å), *T* = 100 K, μ = 1.229 mm^–1^, ρ_calc_ = 1.607 g cm^–3^, 8299 reflections measured (9.7 ≤ 2θ ≤ 140.2°), 1970 unique (*R*_Int_ = 0.038), *R*(*F*) = 0.031 [1804 reflections with *I* > 2σ(*I*)], *wR*(*F*^2^) = 0.078 (all data), flack absolute structure parameter 0.08 (11), Δρ_min,max_ (*e* Å^–3^) = –0.17, +0.17, CCDC deposition number 2362028.

## 4. Conclusions

Further chemistry is developed for substrates with two halogens activated for nucleophilic displacement by strong electron withdrawing groups. The use of compound **2** gave dinitrated heterocyclic benzodioxin **13**, dithiin **14,** and phenoxathiine **15**. All three heterocycles gave satisfactory X-ray single-crystal structure data, but phenoxathiine **15** was disordered. The cyclic thioether of compound **15** was readily oxidised with mcpba to racemic sulfoxide **16** without over-oxidation to the sulfone occurring. The chemistry of this intermediate was not developed [21] any further because of the low yield for the formation of heterocycle **15**. The yield was lower than for the two symmetrical systems **13** and **14**. Presumably, there is a molecular strain in the heterocycle because it is not planar but rather folded with a butterfly shape. As expected, one or two fluorine atoms are easily displaced with compound **2** [3] New aromatic derivatives **23**, **24**, and **25** are reported here. This leaves a further activated fluorine atom and two nitro groups, which both activate each other. A thiol was chosen to react with compound **23** because it is polarisable owing to its large size, making it a good nucleophile. The F atom was displaced, followed by the most reactive nitro group. This gave an interesting 1,4-bis(thiophenyl)benzene derivative **26**, which might be a new reaction not requiring metallic catalysis such as Pd, Pd(II), or Cu(II).

## Data Availability

Aberdeen University Library.

## Supplementary Materials

The following supporting information can be downloaded at: https://www.mdpi.com/article/10.3390/ijms25158162/s1.
