# Supplementary material for: Sequential Nucleophilic Aromatic Substitution Reactions of Activated Halogens
Source: Int J Mol Sci. 2024 Jul 26;25(15):8162. doi: 10.3390/ijms25158162 (PMC11311403; doi:10.3390/ijms25158162)
Supplement: Supplementary file 1 [file ijms-25-08162-s001.zip › ijms-3094836-supplementary.pdf]

Supplementary

Sequential nucleophilic aromatic substitution reactions of activated halogens
M. John Plater* and William T. A. Harrison
Department of Chemistry, University of Aberdeen, Meston Walk, Aberdeen, AB24 3UE
m.j.plater@abdn.ac.uk

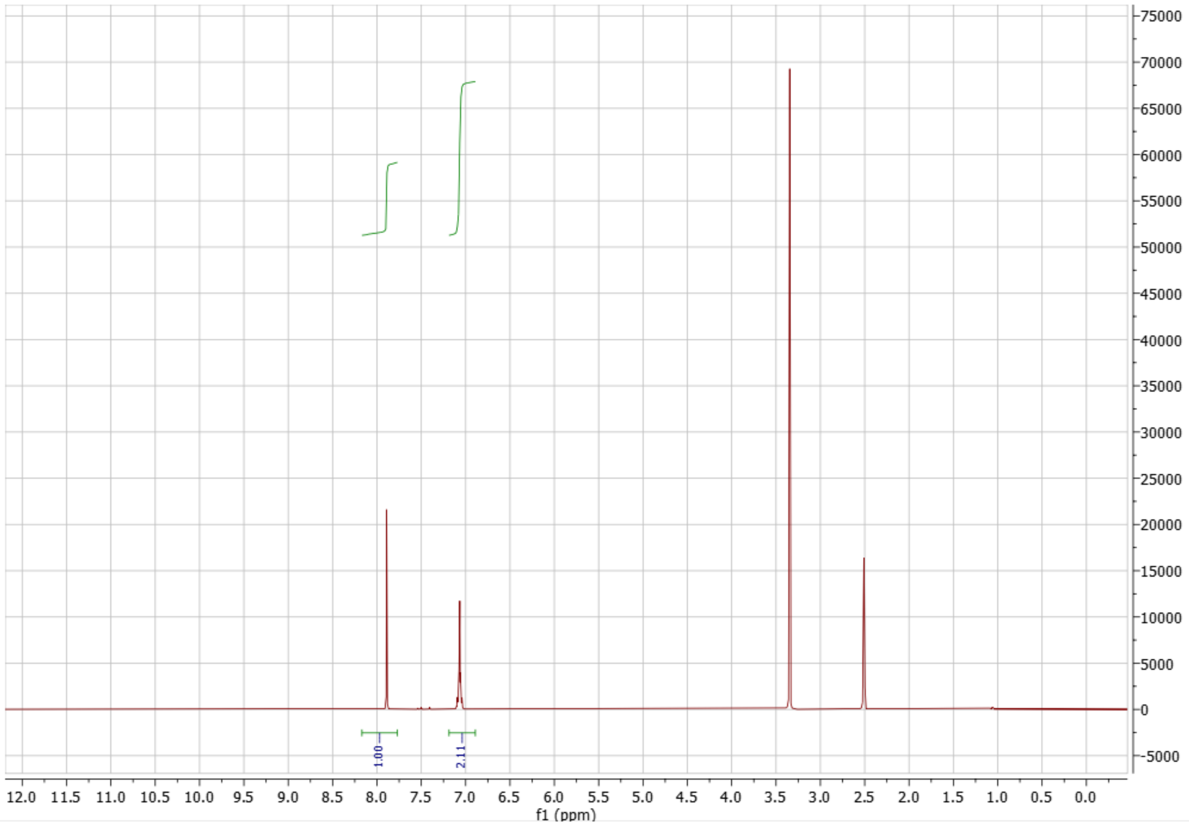

Compound 13 400 MHz Proton NMR D_6DMSO

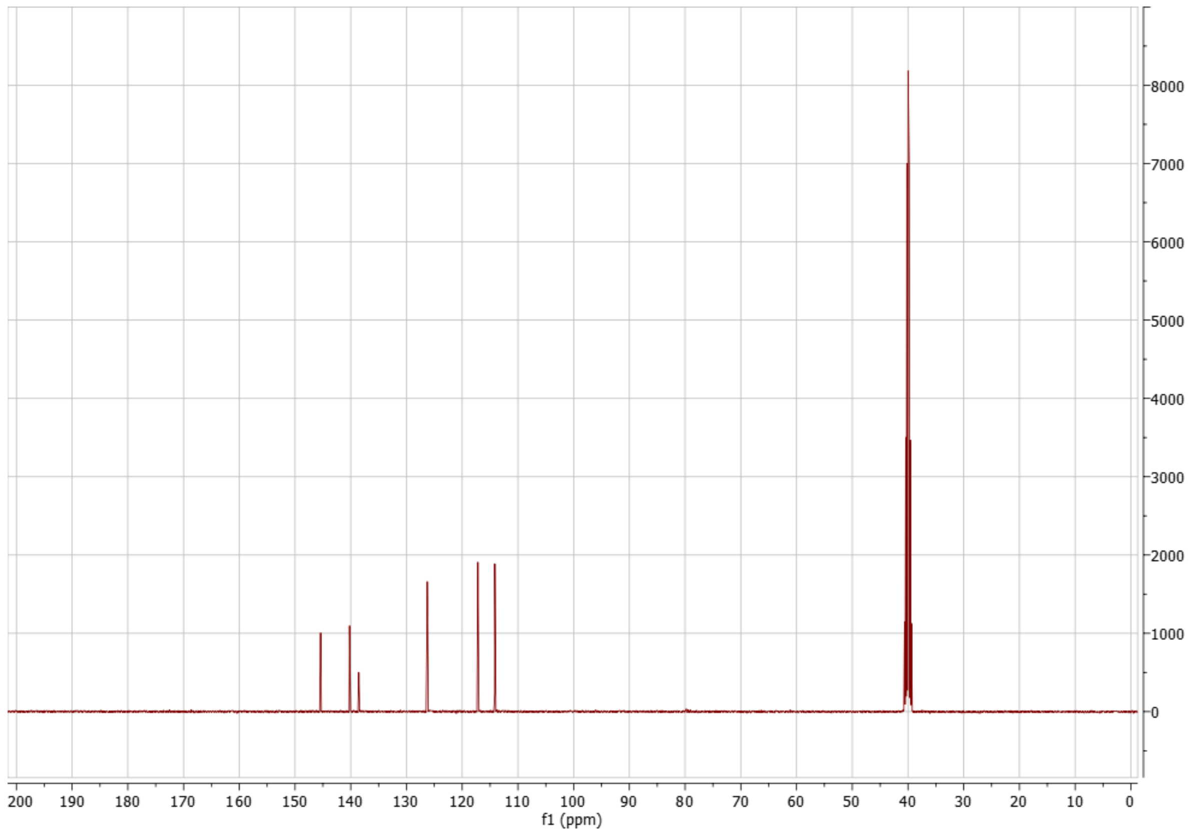

Compound 13 400 MHz 13 Carbon NMR D_6DMSO

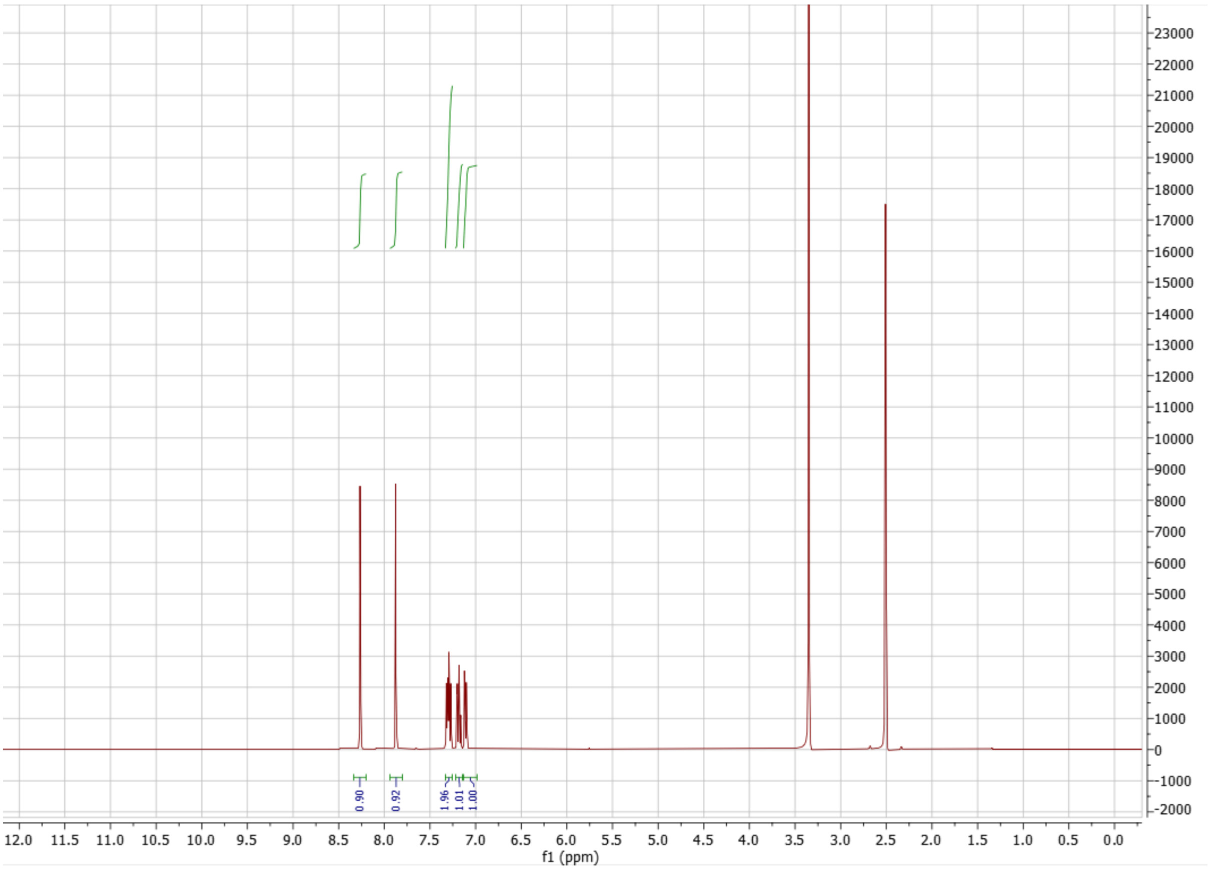

Compound 15 400 MHz Proton NMR D_6DMSO

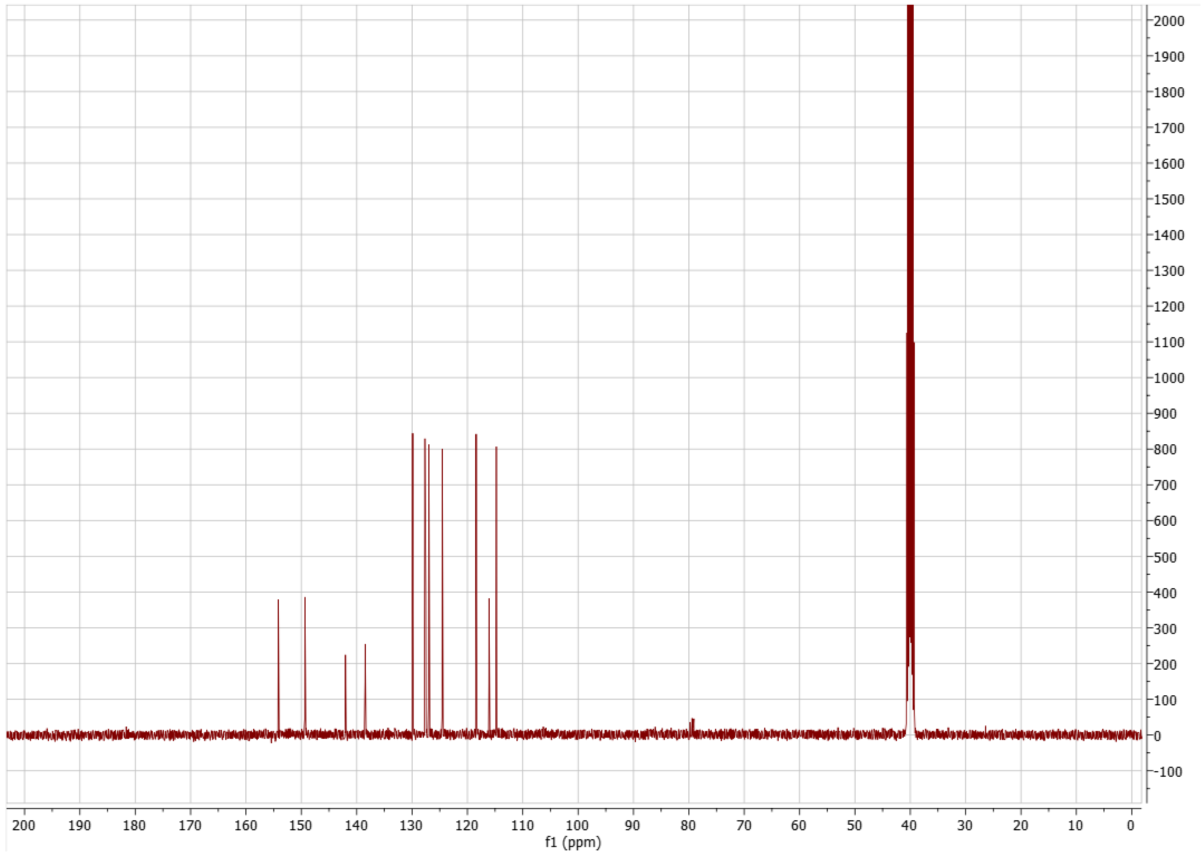

Compound 15 400 MHz 13 Carbon NMR D_6DMSO

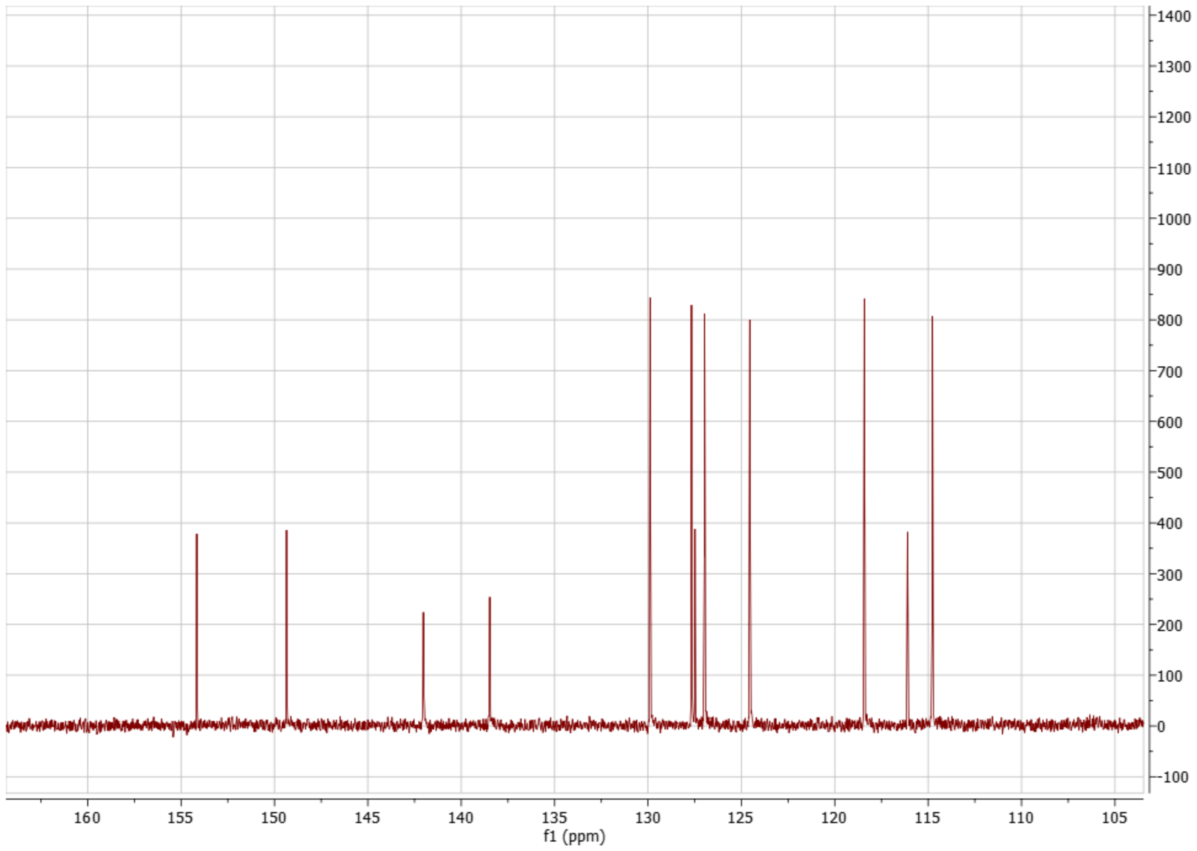

Compound 15 400 MHz 13 Carbon NMR D_6DMSO Expansion

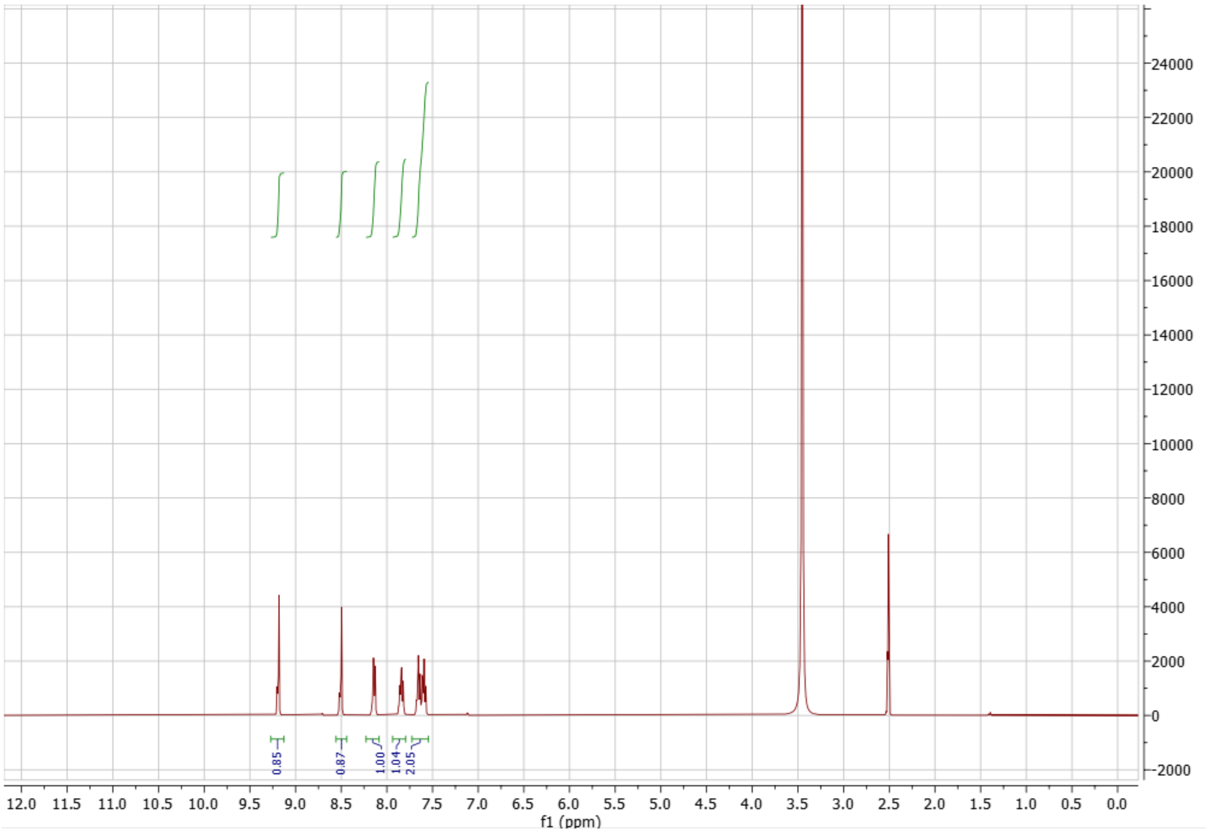

Compound 16 400 MHz Proton NMR D_6DMSO

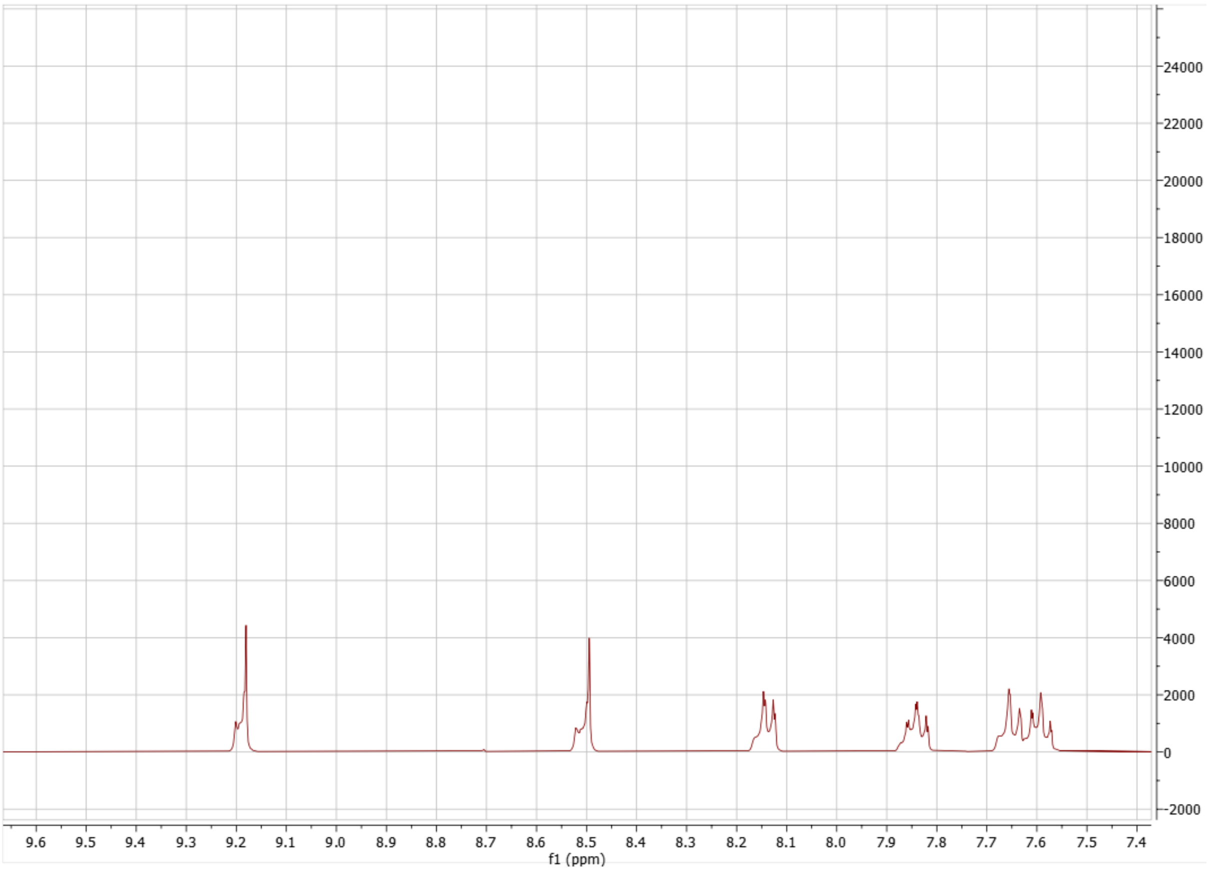

Compound 16 400 MHz Proton NMR D_6DMSO Expansion

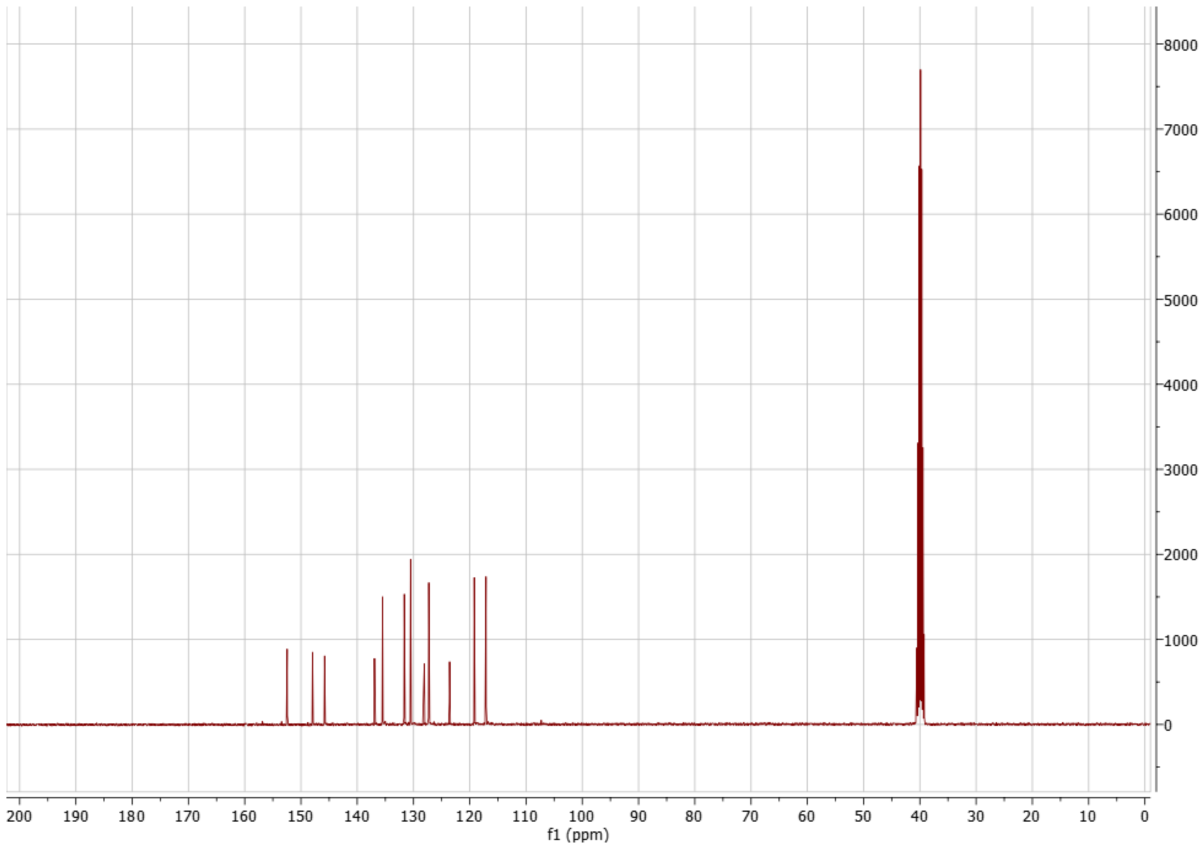

Compound 16 400 MHz 13 Carbon NMR D_6DMSO

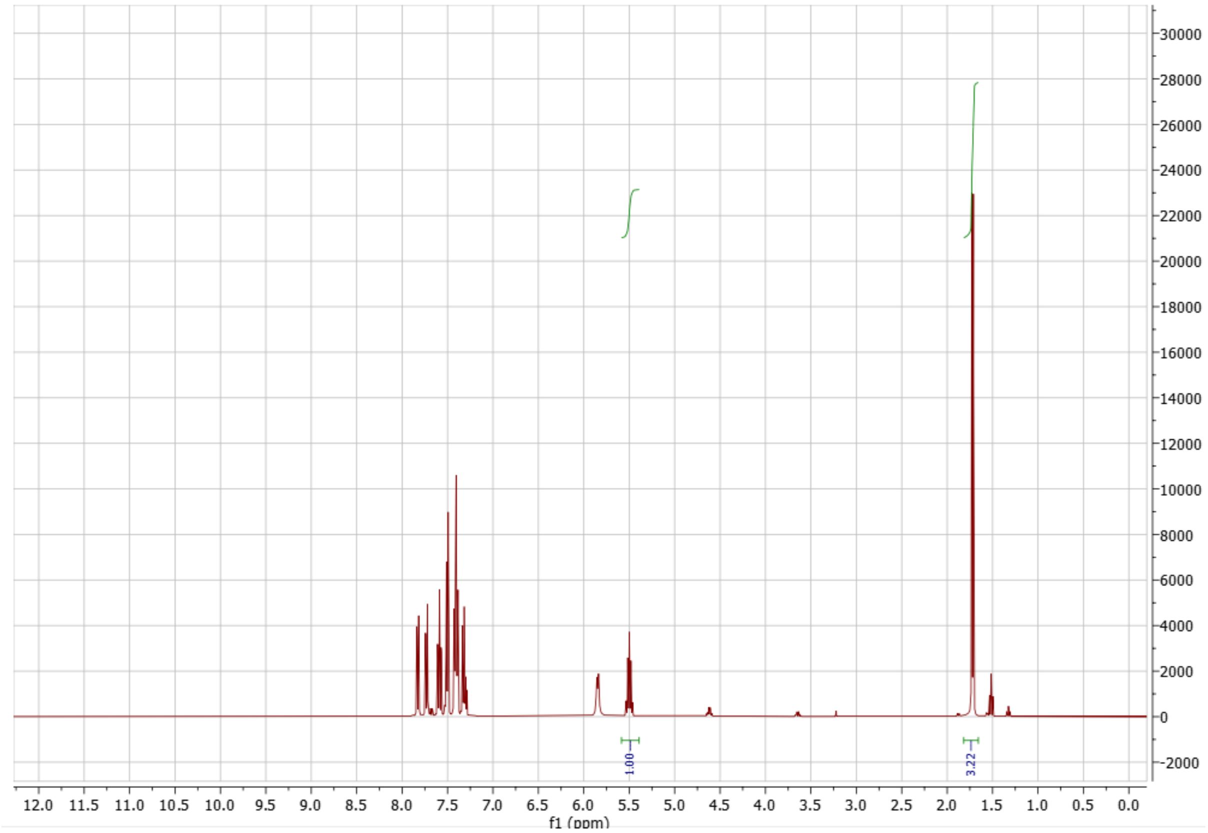

Compound 17 400 MHz Proton NMR $CDCl_3$

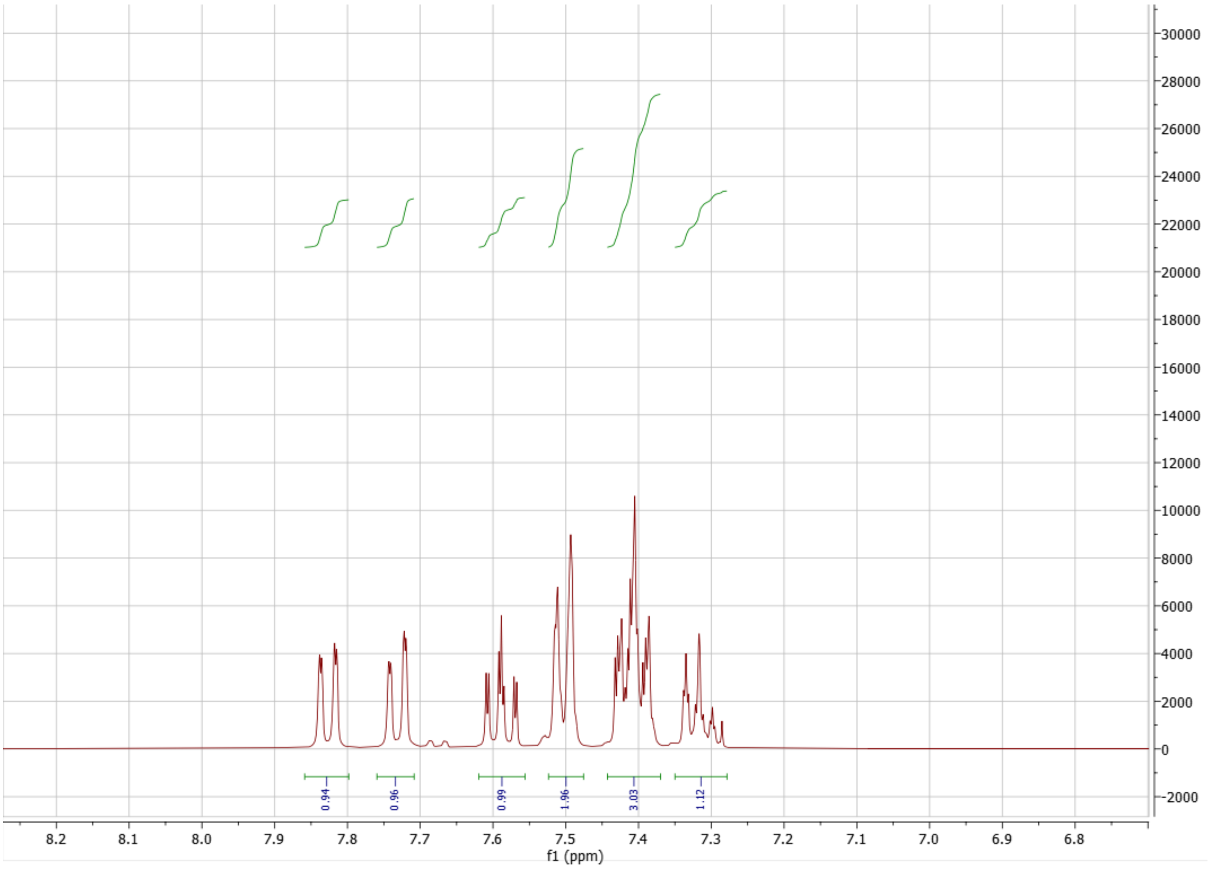

Compound 17 400 MHz Proton NMR $CDCl_3$ Expansion

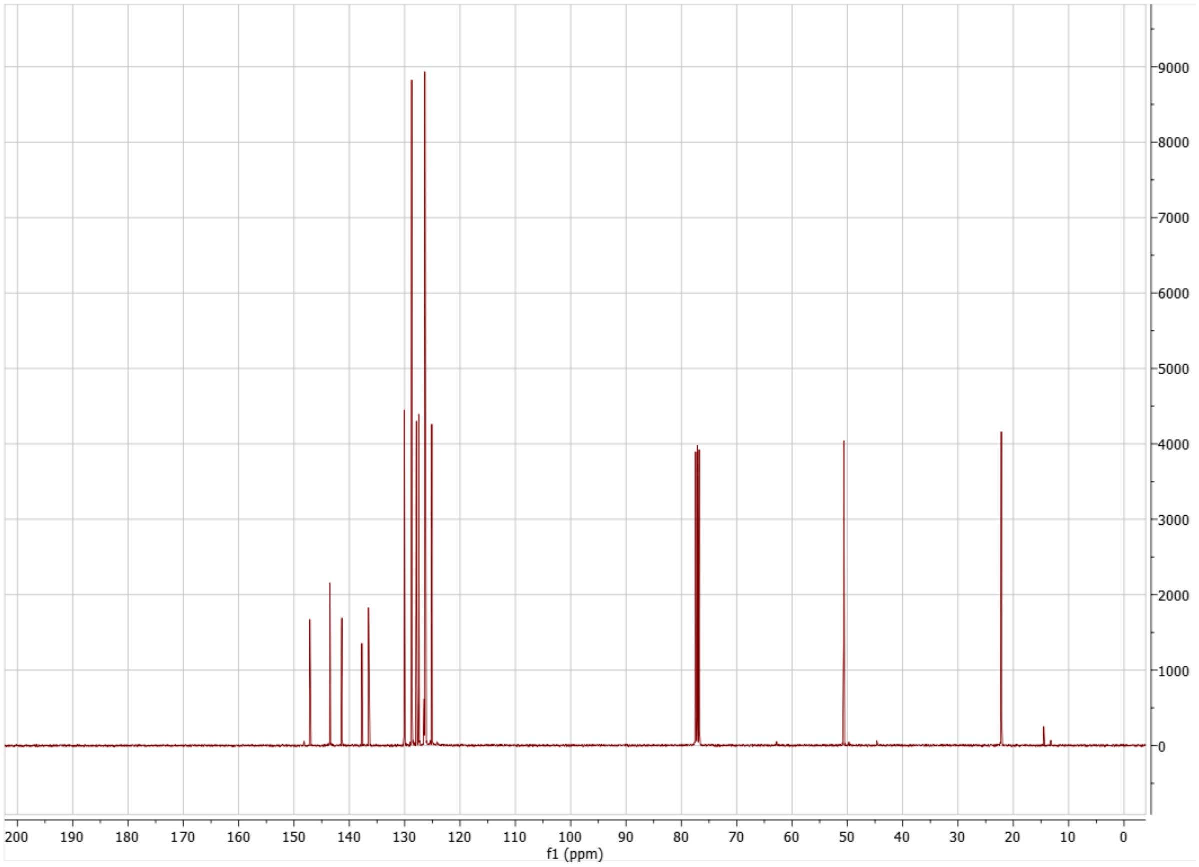

Compound 17 400 MHz 13 Carbon NMR $CDCl_3$

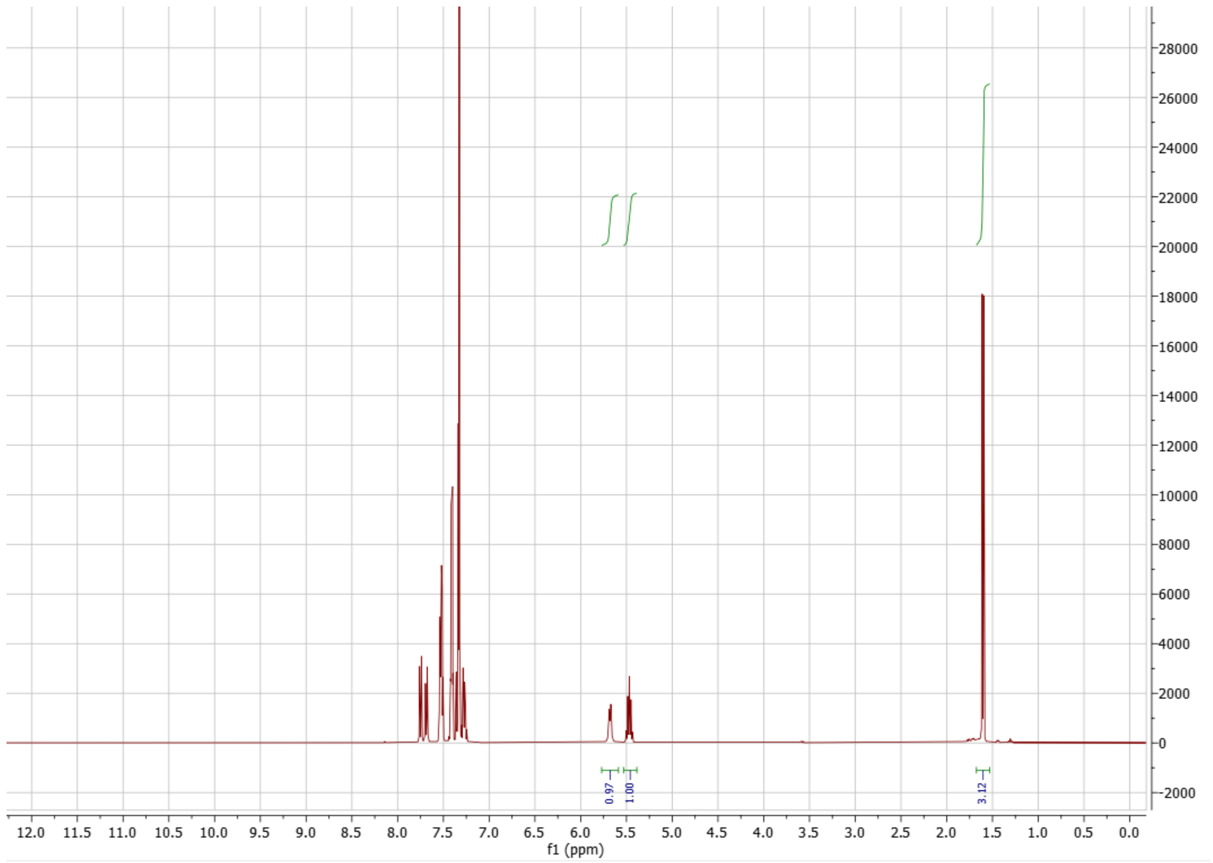

Compound 18 400 MHz Proton NMR $CDCl_3$

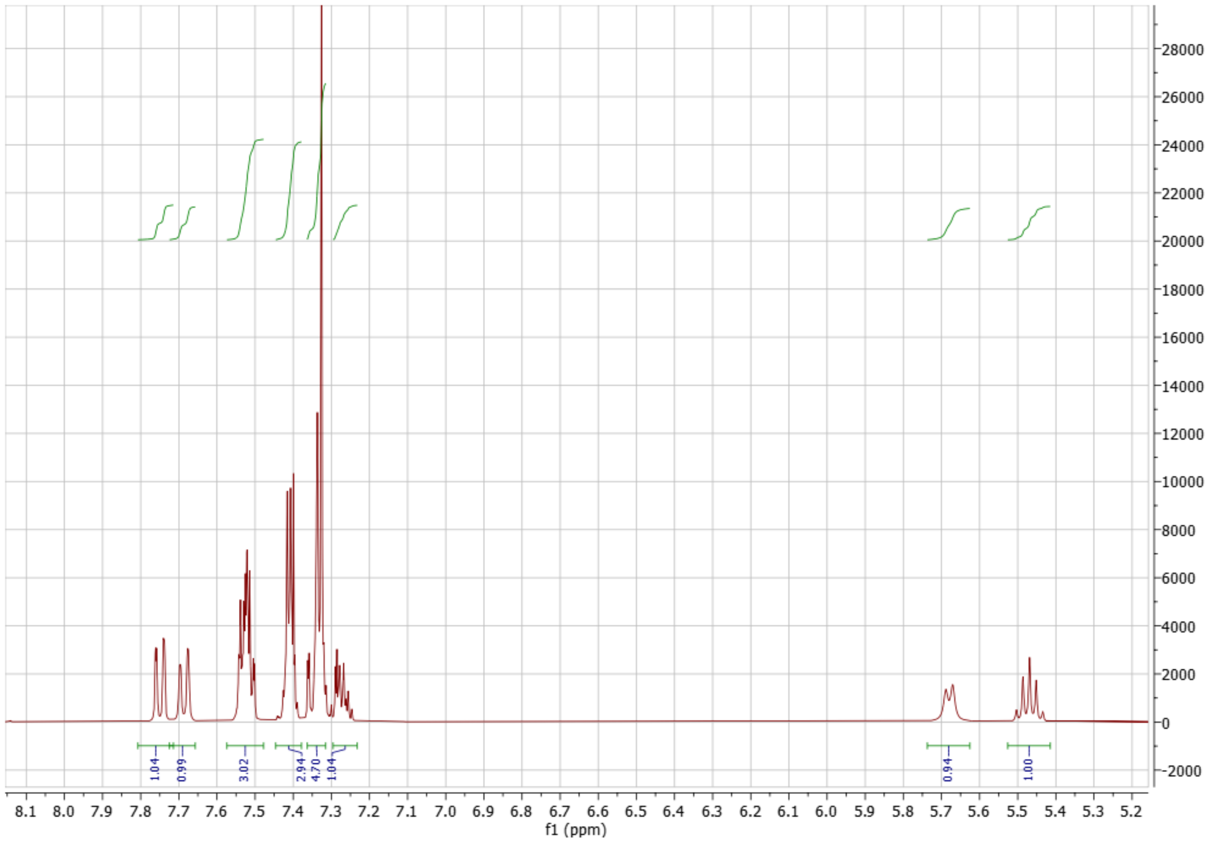

Compound 18 400 MHz Proton NMR $CDCl_3$ Expansion

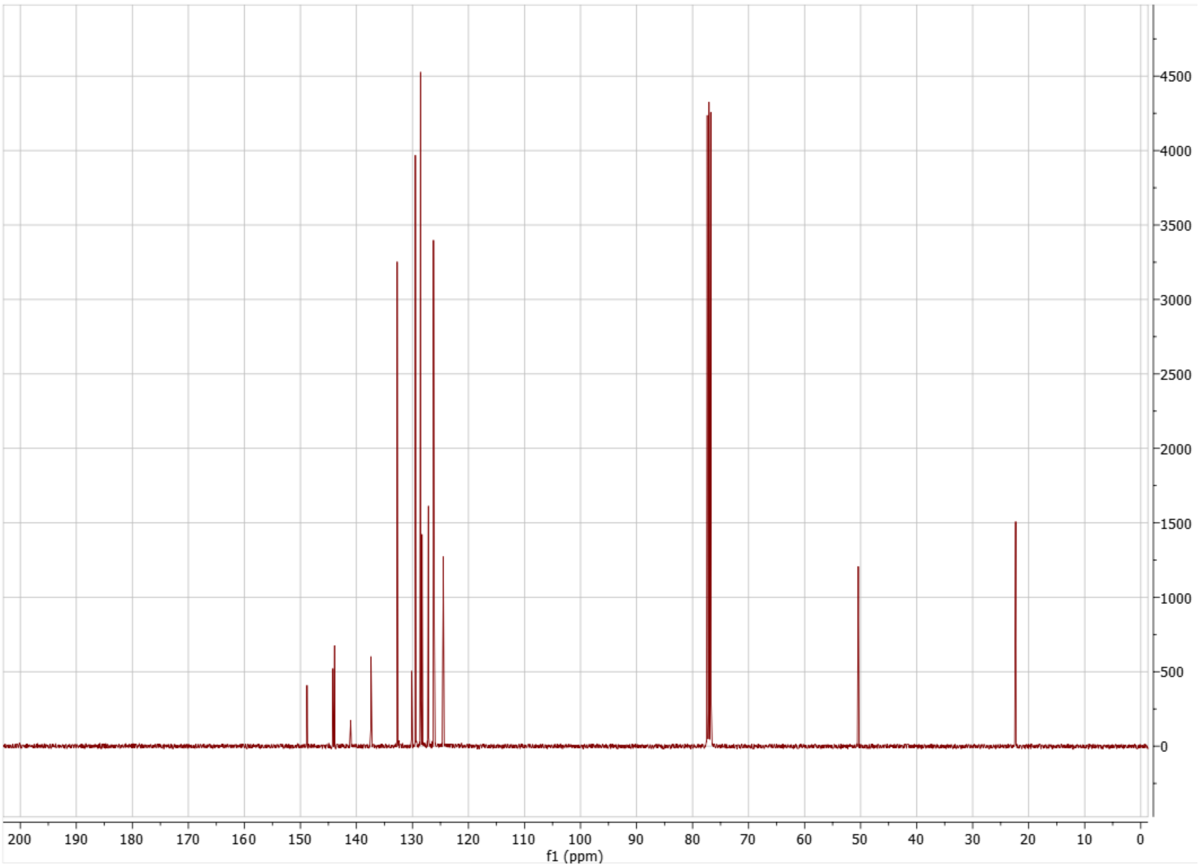

Compound 18 400 MHz 13 Carbon NMR $CDCl_3$

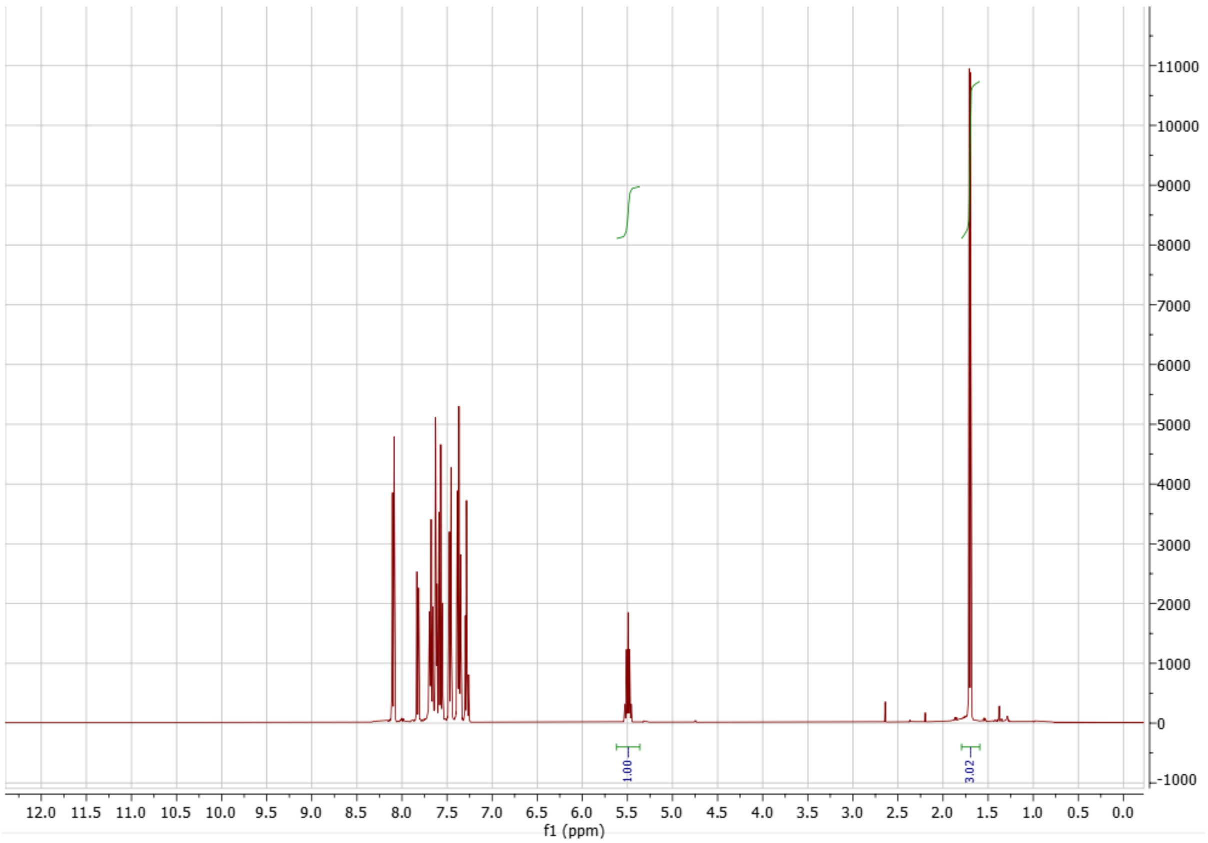

Compound 19 400 MHz Proton NMR $CDCl_3$

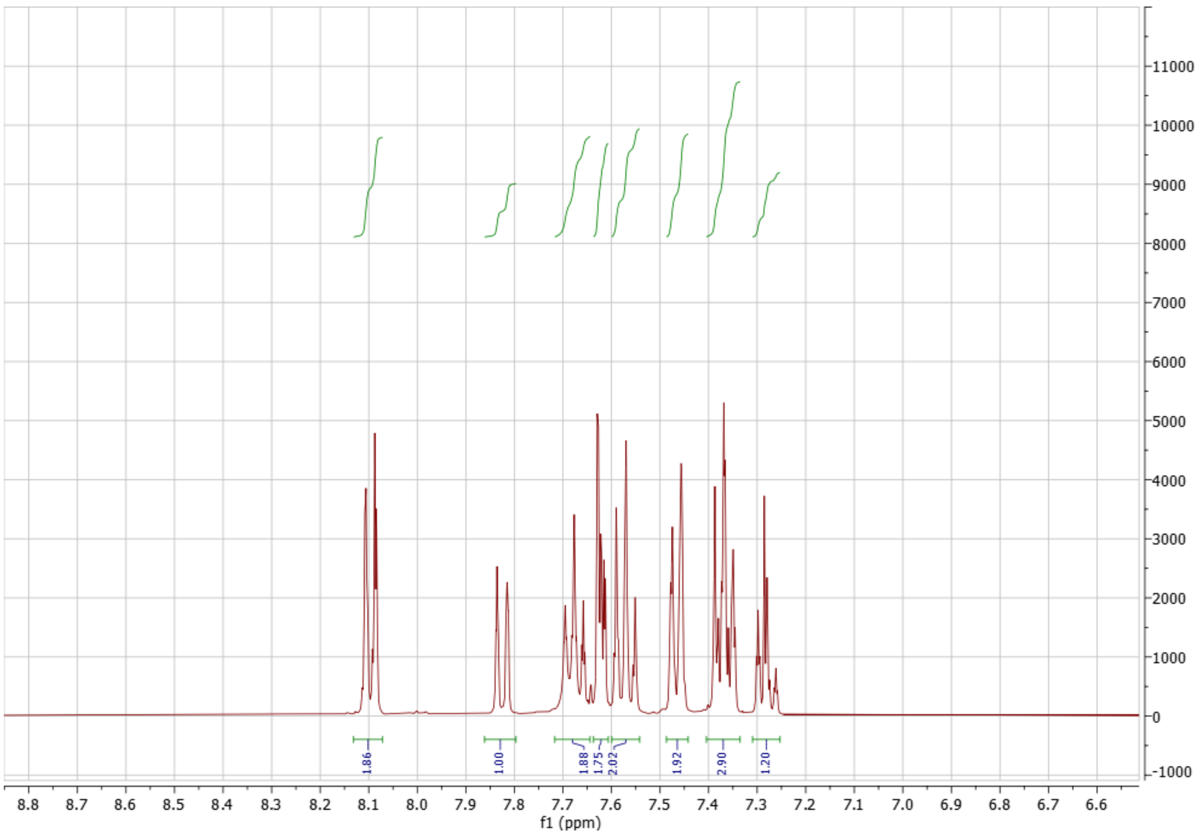

Compound 19 400 MHz Proton NMR $CDCl_3$ Expansion

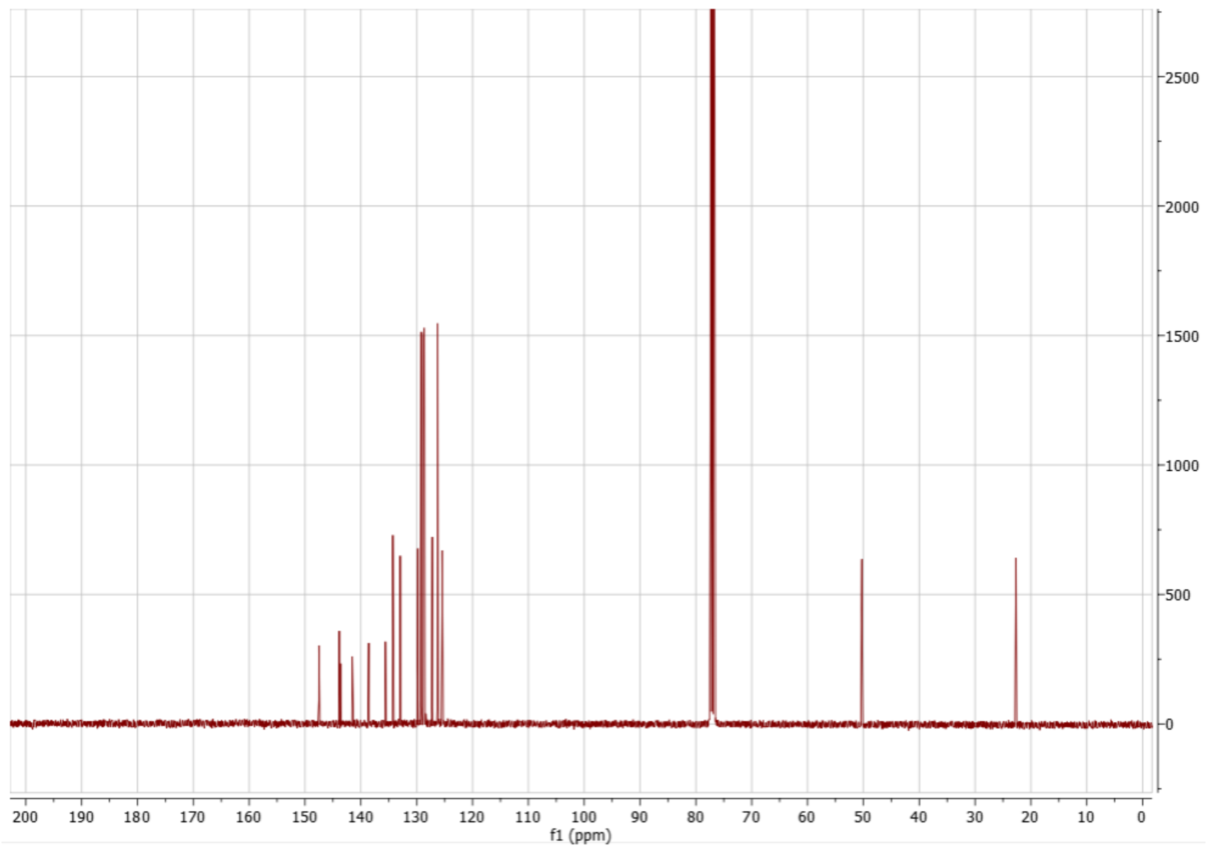

Compound 19 400 MHz 13 Carbon NMR $CDCl_3$

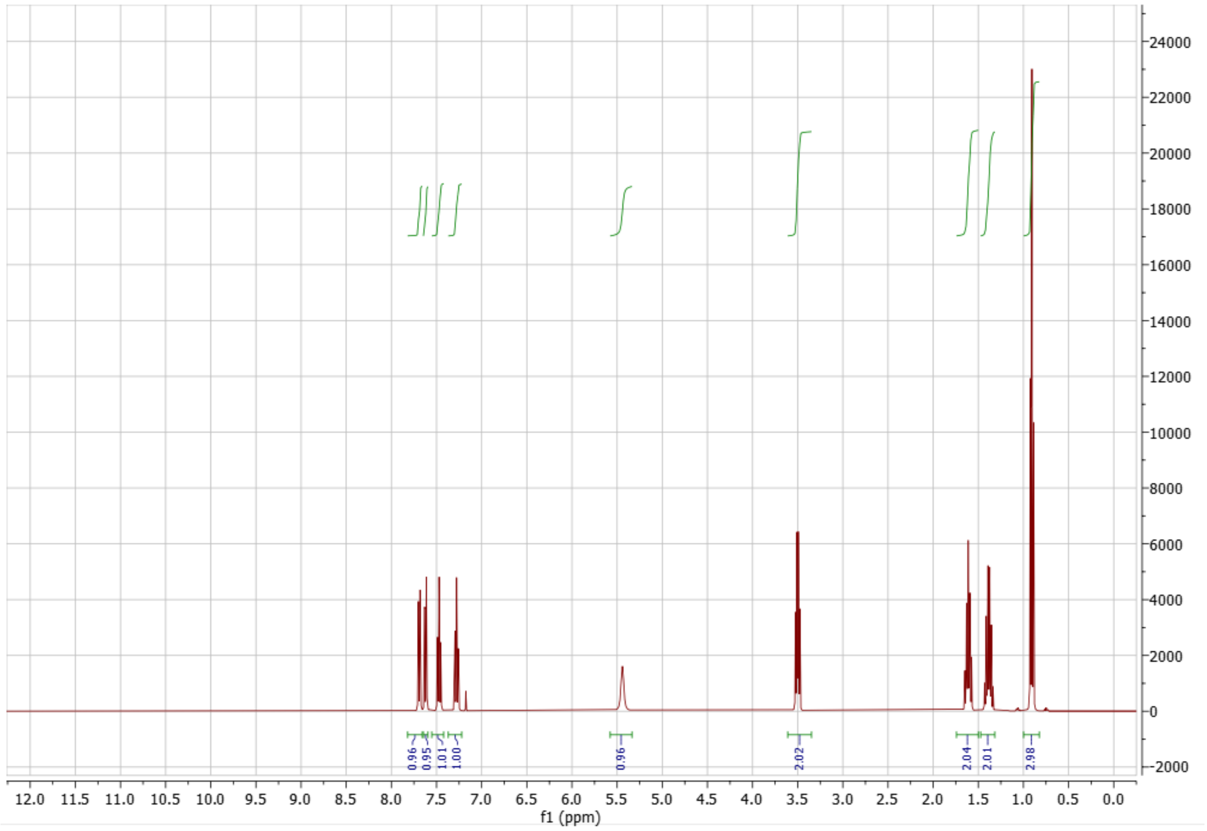

Compound 20 400 MHz Proton NMR

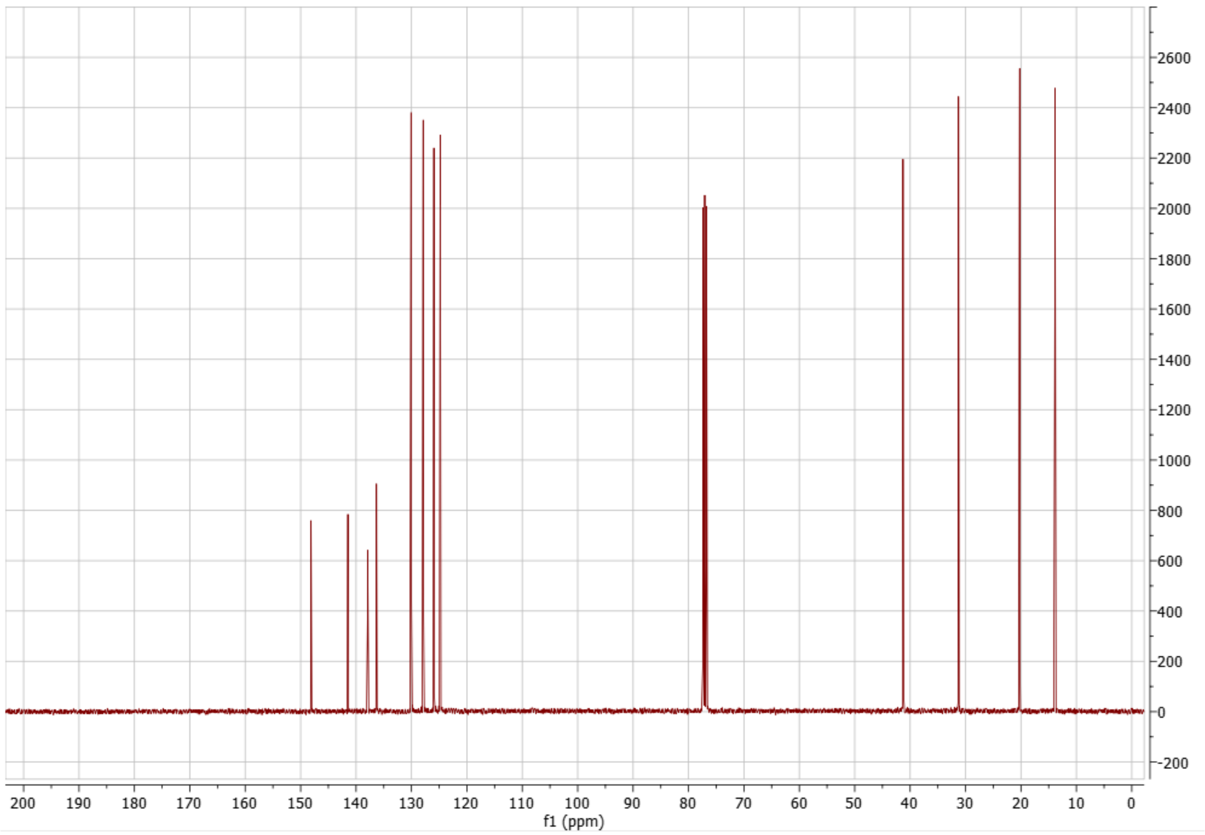

Compound 20 400MHz 13 Carbon NMR

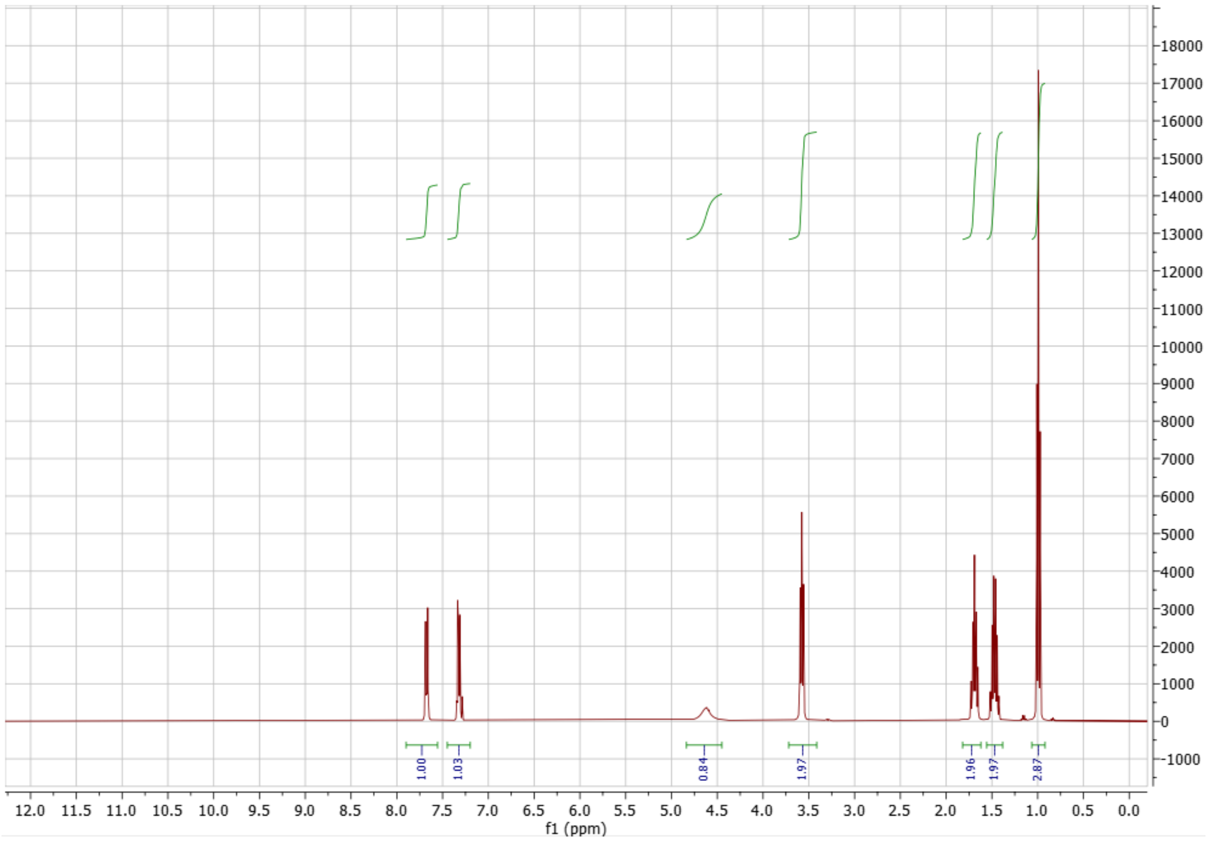

Compound 21 400MHz Proton NMR

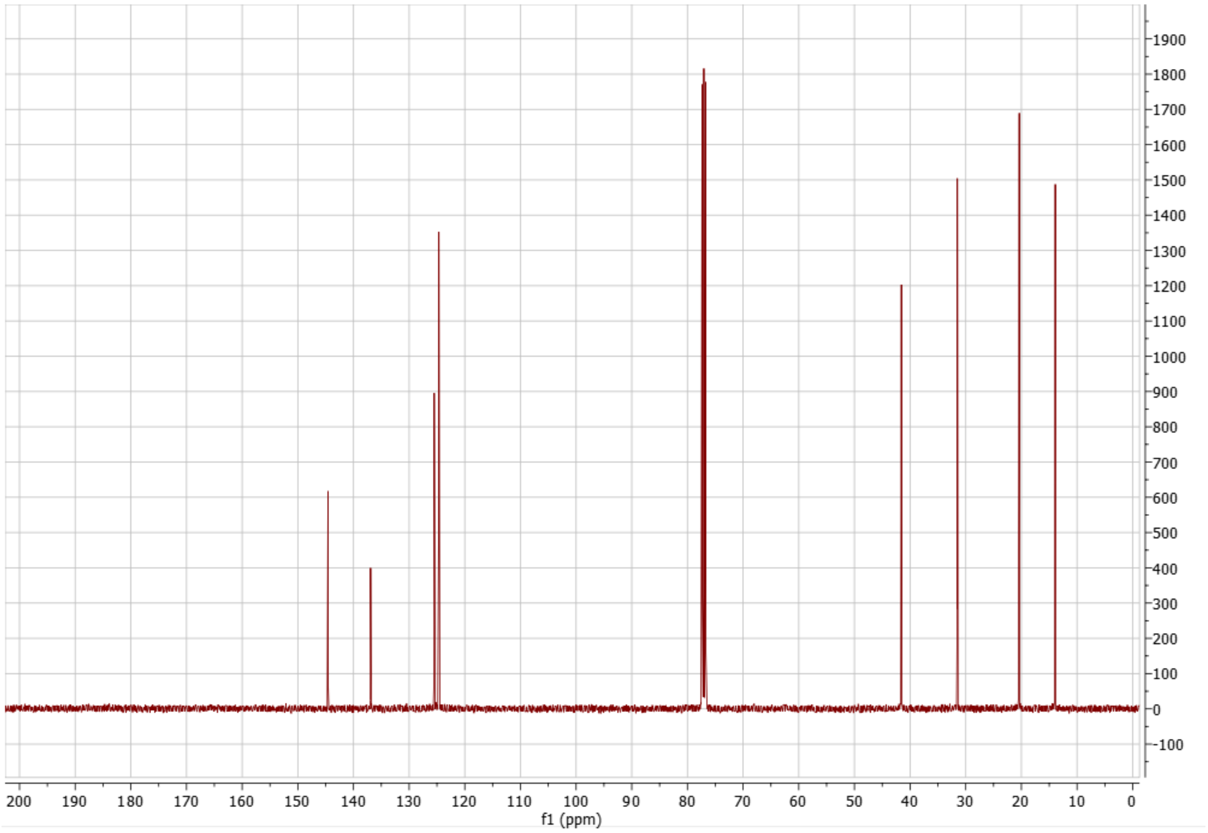

Compound 21 400 MHz 13 Carbon NMR

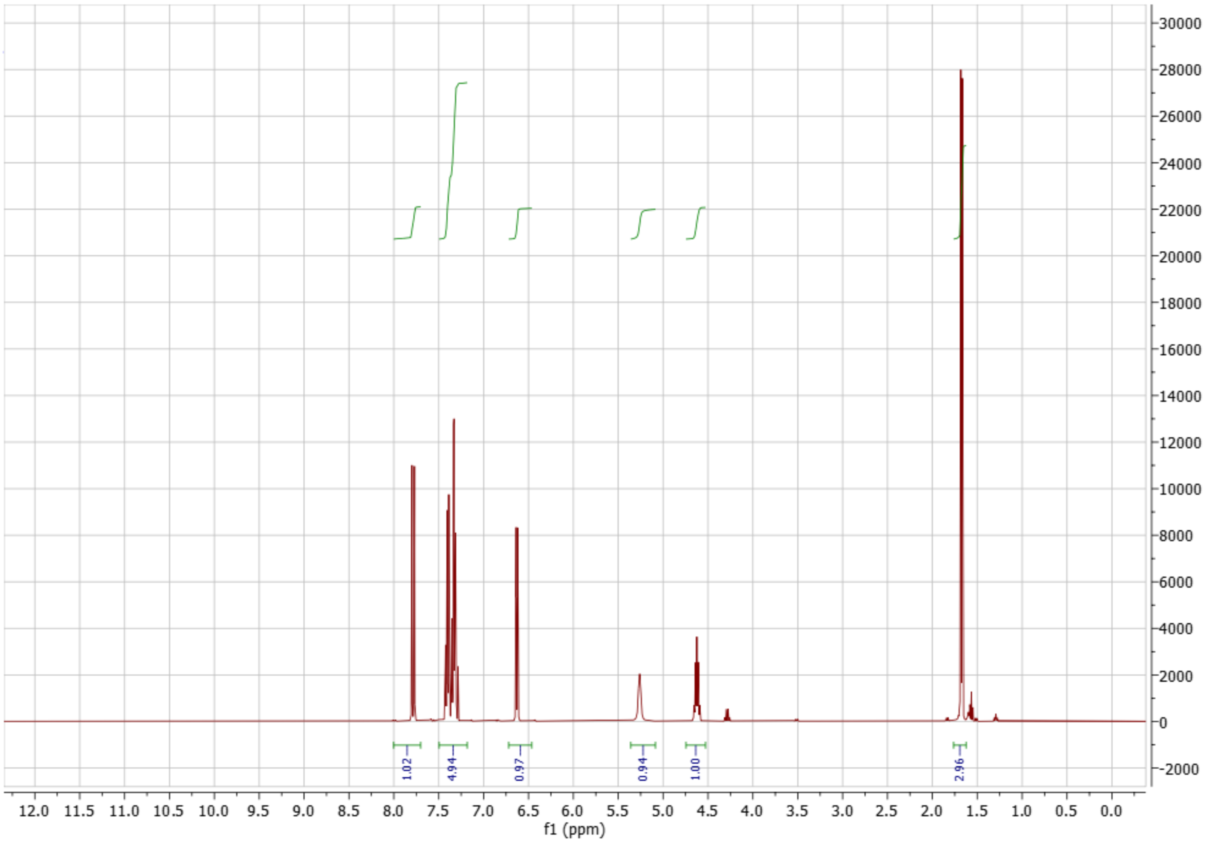

Compound 23 400 MHz Proton NMR $CDCl_3$

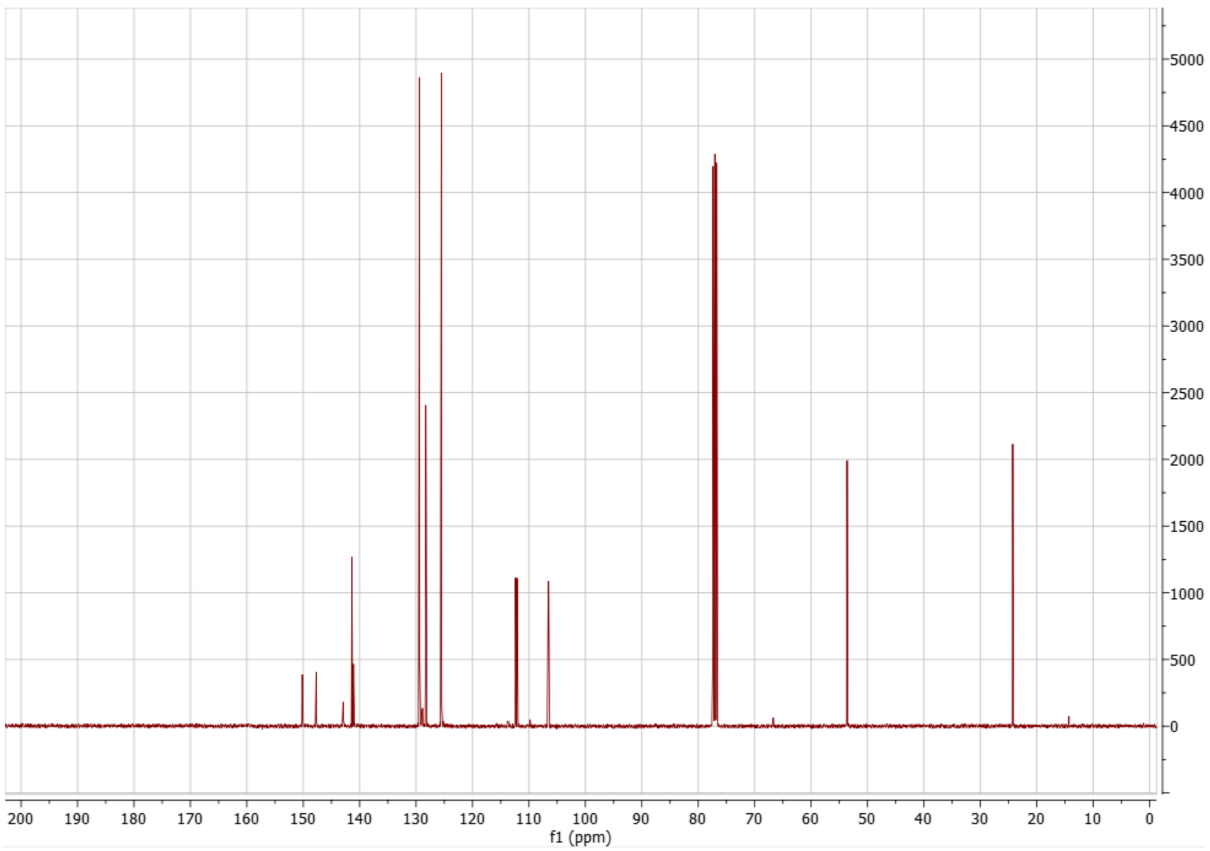

Compound 23 400 MHz 13 Carbon NMR $CDCl_3$

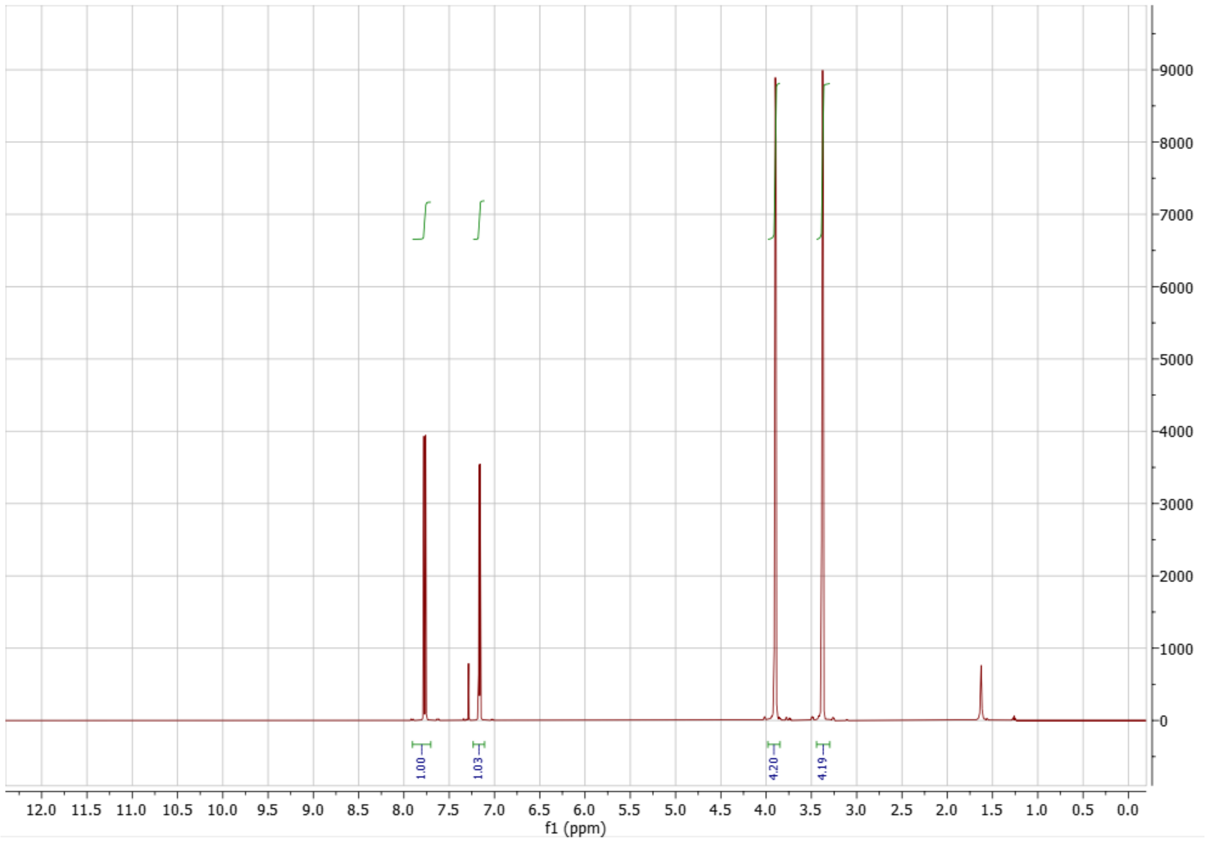

Compound 24 600 MHz Proton NMR $CDCl_3$

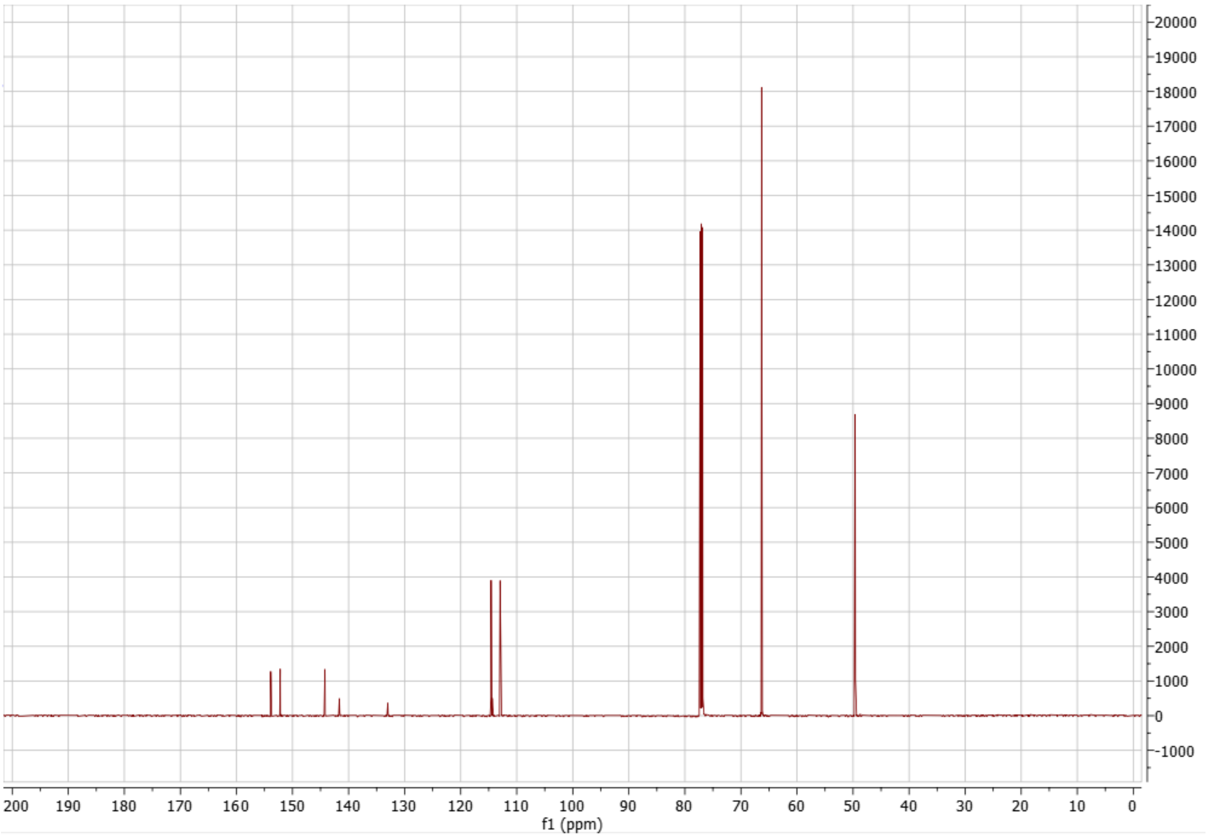

Compound 24 600 MHz 13 Carbon NMR $CDCl_3$

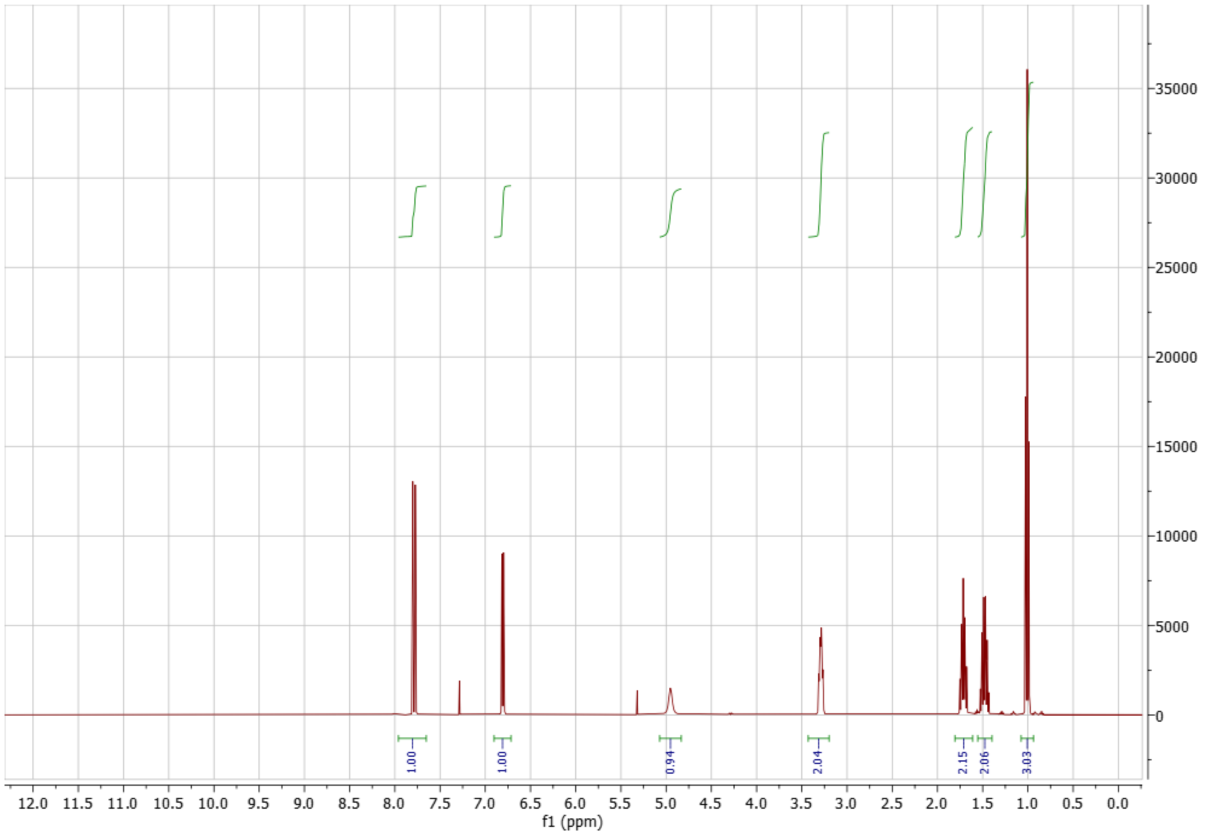

Compound 25 400 MHz Proton NMR $CDCl_3$

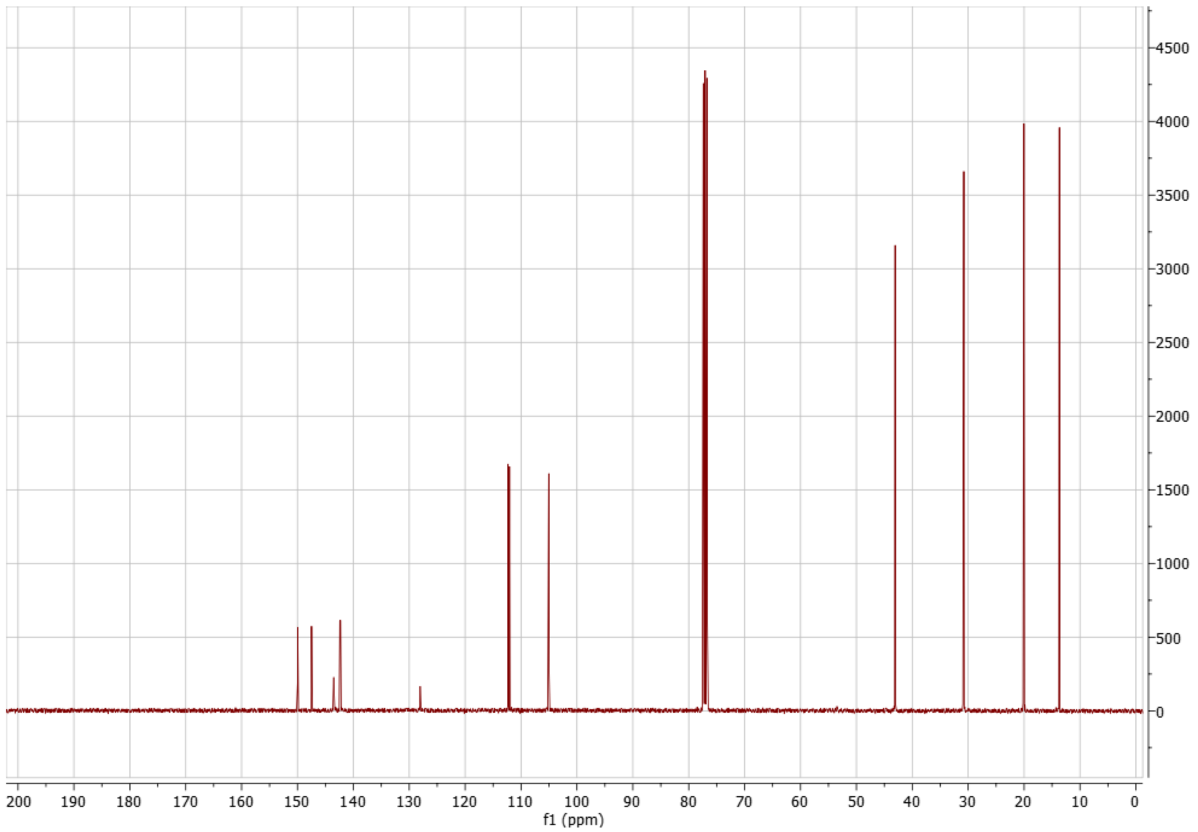

Compound 25 400 MHz 13 Carbon NMR CDCl$_3$

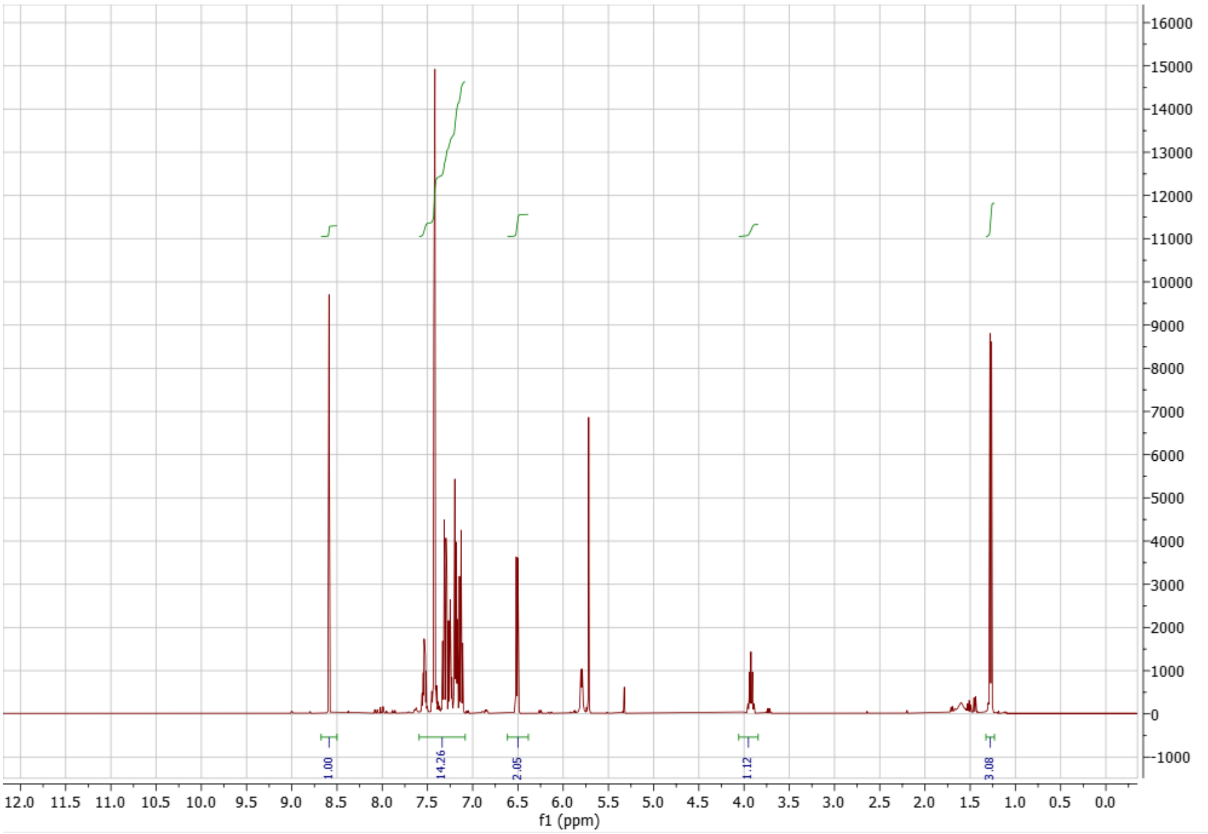

Compound 26 400 MHz Proton NMR $CDCl_3$

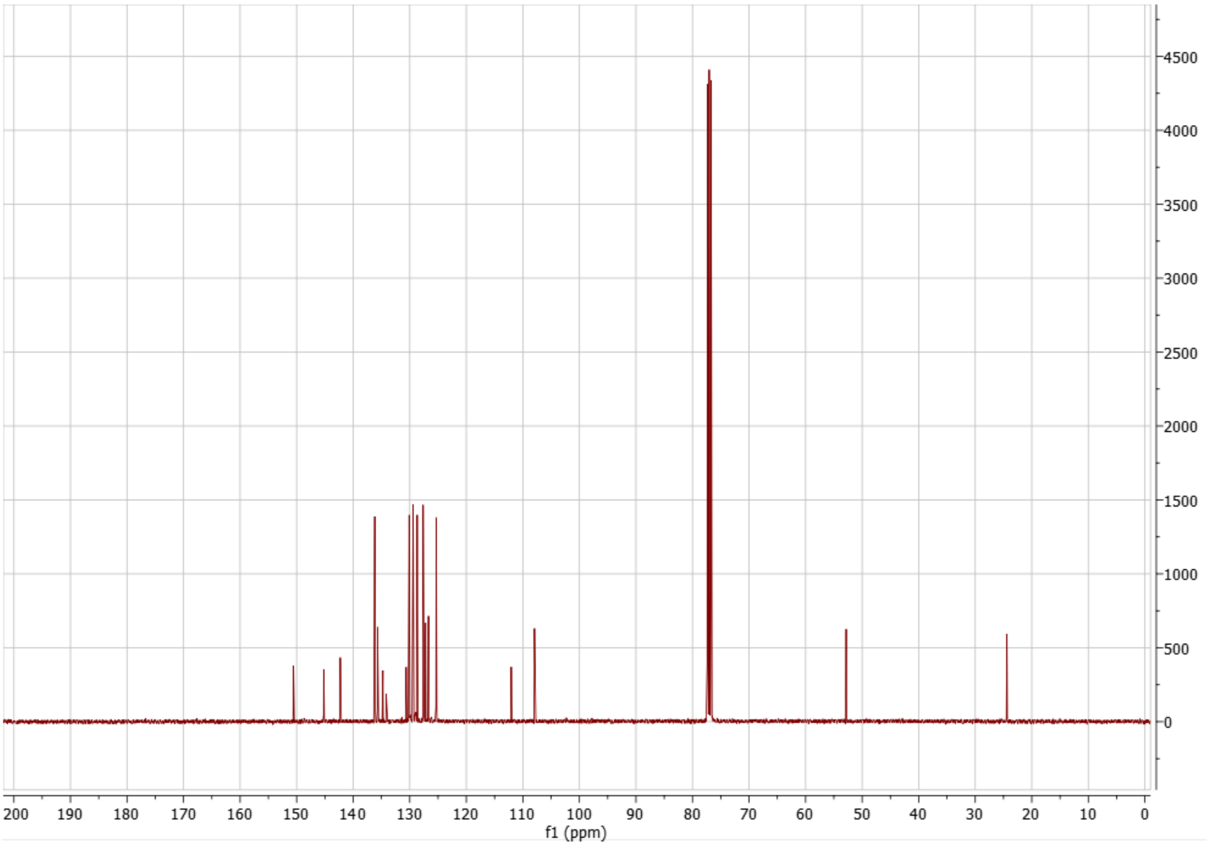

Compound 26 400 MHz 13 Carbon NMR $CDCl_3$
